# Phylogeny and systematics of the colubrid snake genera *Liopeltis* and *Gongylosoma* (Squamata: Colubridae) and description of a new Himalayan endemic genus and species

**DOI:** 10.1038/s41598-024-74271-1

**Published:** 2024-10-21

**Authors:** Zeeshan A. Mirza, Virender K. Bhardwaj, Saunak Pal, H. T. Lalremsanga, Gernot Vogel, Patrick D. Campbell, Harshil Patel

**Affiliations:** 1 Max Planck Institute for Biology, Max-Planck-Ring 1, 72076 Tübingen, Germany; 2 Developmental Biology and Herpetology Laboratory, Department of Zoology, Mizoram University, Aizawl, 796004 Mizoram India; 3 School of Natural and Environmental Sciences, Newcastle University, Newcastle Upon Tyne, NE1 7RU UK; 4 Society for Southeast Asian Herpetology, Heidelberg, Germany; 5 Science Department, The Natural History Museum, London, SW7 5BD UK; 6 Thackeray Wildlife Foundation, Mumbai, India

**Keywords:** Cryptic species, MicroCT, Phylogeny, Reptilia, Serpentes, Taxonomy, Herpetology, Phylogenetics, Taxonomy

## Abstract

The colubrid snakes of the genera *Gongylosoma* Fitzinger, 1843 and *Liopeltis* Fitzinger, 1843 are distributed across south and southeast Asia with five and eight nominate species, respectively. Despite their wide distribution, members of these genera are among some of the least-known colubrids. The two genera were considered synonymous in the past only to be separated later, and are defined on rather nebulose characters with a lack of support from molecular data. To test the monophyly of the two genera, we generated molecular data for the type species of *Gongylosoma* and species representing the two genera, including samples of *Liopeltis rappii* (Günther, 1860) from the western Himalayas. Results recovered paraphyly of *Liopeltis*, especially with regard to the genus *Gongylosoma.* Morphological data supports recognizing the western and eastern populations of *L. rappii* as two distinct species. The findings from our integrative taxonomic approach advocate establishing a new genus to embody *Liopeltis rappii* and a new allied species from the central and western Himalayas. A rediagnosis and revised classification of the genera *Gongylosoma* and *Liopeltis* is presented. The results further hint at cryptic diversity across members of the two genera, warranting scrutiny of the most widespread members of the group.

**Supplementary Information:**

The online version contains supplementary material available at 10.1038/s41598-024-74271-1.

## Introduction

Members of the family Colubridae are diverse serpents distributed across all continents except for Antarctica^1^. Due to the wide distribution of the family, it has been a subject of interest with regard to the phylogenetic relationship within genera across the family^2–4^. However, the lack of molecular data for type species of some genera and missing taxa are a major impediment in inferring robust phylogenies and consequential taxonomic amendments. Furthermore, evolutionary analyses of the dynamics of speciation, extinction and phenotypic evolution are also hindered. One such group are snakes of the genera *Gongylosoma* Fitzinger, 1843 and *Liopeltis* Fitzinger, 1843. These snakes are generally small-sized in relation to other members of the family and are ground-dwelling diurnal snakes^5,6^. Certain species of the two genera are widespread, while others are known from either the type specimens or a few isolated localities^7–10^.

The genera *Liopeltis* and *Gongylosoma* were described to accommodate *L. tricolor* (Schlegel, 1837) and *G*. *baliodeira* (Boie, 1827). Currently, eight species represent the genus *Liopeltis*, and *Gongylosoma* by five species, distributed across South and Southeast Asia^7,8,11^. In the past, most current members of the two genera had been earlier assigned to the genus *Ablabes* Dumeril, 1853^12,13^. The members have had a complex taxonomic history, with several nomenclatural changes and species assigned to various genera in the late 19th century to mid-20th century^6,9,14^. Leviton^9^, split the then-existing members of the genus *Liopeltis* based on diagnostic characters and revalidated the genus *Gongylosoma*^3,9,11,15^. Nearly all members of the genus *Liopeltis* and *Gongylosoma* are poorly known, as they are small and difficult to encounter on surveys, evidenced by the paucity of specimens of some of the species in natural history museums^3,6,8,16^. A recent study by Lalremsanga et al.^17^ showed that *Gongylosoma scriptum* (Theobald, 1868) is embedded within the genus *Liopeltis*. However, Lalremsanga et al.^17^ refrained from making taxonomic changes as molecular data for the type species of the genus *Gongylosoma*, *i.e. G. baliodeira* and several other members were unavailable^8^.

Among the eight putative species of the genus *Liopeltis*, *Liopeltis rappii*^18^, which was originally described from Sikkim, India, in the genus *Ablabes*, thus far remains the least known member of the genus. Günther^18^ described *Ablabes rappii* along with *Ablabes owenii*, which was subsequently treated as a junior synonym of the former^6,12^. Since then, the species has been reported from several localities across the Himalayas^6,13,19^, but no new information regarding the systematics of the snake has been published. As part of an ongoing study on Indian colubrid snakes, we reviewed the current knowledge of the species based on existing literature and the re-examination of museum material, including the types, while also paying attention to the osteology of the skull. Furthermore, freshly collected specimens from the Western Himalayas allowed us to elucidate the phylogenetic relationship of the species and those assigned to the genera *Liopeltis* and *Gongylosoma*. The results of the assessment are at odds with the current taxonomy of the two genera and species. The present work proposes a new genus to accommodate *Liopeltis rappii*, an undescribed species, and provides a new classification of the genera *Liopeltis* and *Gongylosoma*.

## Materials and methods

### Morphology

Field collection was performed with permission from the Ministry of Environment, Forest and Climate Change, Government of Himachal Pradesh, and the study was approved by the Institutional Ethics Committee of Mizoram University. The methods were performed in accordance with the local legislation and institutional guidelines. The study is based on 30 specimens representing *L. rappii*, fifteen of these specimens originate (including the holotypes of *L. rappii* and *L. owenii*) from the eastern Himalayas, and the other fifteen specimens from the western Himalayas. Two of the specimens from the western Himalayas (Himachal Pradesh) were caught, photographed, and later euthanized using Halothane as per standard euthanasia protocol for reptiles^20^. A third specimen was killed by locals and subsequently cleaned and preserved. Descriptions and mensural characters were compared with the available literature^6–8^. The dorsal scale rows were counted at the first ventral, midbody, and last ventral. Subcaudal scale counts do not include the terminal scute. Values for symmetric head characters are given in right-to-left order. Description style follows Patel et al.^21^ and Mirza et al.^22^ with some modifications. Scalation and other comparable characters are described as ventral scales (VS); subcaudal scales (SC); dorsal rows of scales (DSR); supralabial scales (SL); loreal scales (LS); preocular scales (PreO); postocular scales (PostO); temporal scales (T); infralabial scales (IL); snout-vent length (SVL); tail length (TaL); total length (TL); head length (HL); head width (HW). Measurements were taken with the help of a digital caliper to the nearest 0.1 mm, and those for snout to vent length (SVL), and tail length (TaL) were taken with the aid of a piece of string, which was then measured using a scale. Number of scales bordering the posterior border of both parietals were counted (STP, scales touching parietals); these exclude the temporal scales. Multivariate Principal Component Analysis was performed on morphometric and meristic data for members of the genera *Liopeltis* and *Gongylosoma* (Supplementary Table S1). Morphometric values were corrected for SVL prior to the analysis and were log-transformed to normalize the data. Meristic data were used in their original state without log-transformation.

The hemipenis of the freshly euthanized male paratype was everted by palpation of the organ until it was everted to the maximum extent, after which the organ was separated by making an incision around its circumference at the cloacal region and was immersed in warm water (50 °C) for about 5 min to soften the tissue. Then, it was slowly everted using a blunt pair of forceps, gently pushing the organ from the distal to proximal end. After eversion, the organ was inflated with 4% formaldehyde and tied at the base with a thread. Observations were made using a stereomicroscope (Omano OM2360-BL). Descriptions of hemipenal morphology and terminology follow Smith^6^, Dowling and Savage^23^, and Zaher^24^.

Acronyms of institutions where comparative material was examined: BNHS- Bombay Natural History Society, Mumbai, India; CAS- California Academy of Sciences, San Francisco, USA; FMNH- Field Museum of Natural History, Chicago, USA; MCZ- Museum of Comparative Zoology, Cambridge, USA; MNHN- Muséum national d’Histoire naturelle, Paris, France; MZMU- Museum of Zoology, Mizoram University, Aizawl, India; NCBS- Collection Facility of the National Centre for Biological Sciences, Bangalore, India; NHMUK- Natural History Museum, London, U.K.; NHMW- Naturhistorisches Museum Wien, Vienna, Austria; NMB- Naturhistorisches Museum, Basel; RMNH- Naturalis-Nationaal Natuurhistorisch Museum, Leiden; SMF- Senckenberg Naturmuseum, Frankfurt, Germany; ZMMU- Zoological Museum of Moscow University, Moscow, Russia; ZMH- Zoologisches Institut und Museum, Universität Hamburg, Germany; ZFMK- Zoologisches Forschungsmuseum Alexander Koenig, Bonn, Germany; ZMB- Museum für Naturkunde, Berlin, Germany; ZSI/HARC- Zoological Survey of India, High Altitude Regional Centre, Solan India; ZSIK- Zoological Survey of India, Kolkata, India. LSID for this publication: urn:lsid:zoobank.org:pub:DE27D881-860B-4362-A669-479F67EDDC19.

Micro-CT scan of the skulls were generated for *Liopeltis rappii* (BNHS 743), a male specimen from the Himachal Pradesh population (NCBS NRC-AA-0013), *L. calamaria* (BNHS 666), and *Gongylosoma scriptum* (MZMU 2041) (Supplementary Figure S1, S2 using a Bruker^®^ Skyscan 1272 (Bruker BioSpin Corporation, Billerica, Massachusetts, USA). The head of each of the specimens was scanned for 210 min at a resolution of 5.4 μm and recording data for every 0.4° rotation for 360° with (AL) 1 mm filter. The source voltage for the scan was 65 kV, and source current was 153 µA. Volume rendering was performed with CTVox (Bruker BioSpin Corporation, Billerica, Massachusetts, USA), and images were edited in Adobe Photoshop CS6. Osteological description is based on volume renders retrieved from CTVox following terminology of the skull described by Heatwole^25^.

## Molecular analysis

In order to resolve the phylogenetic position of the focal species, *Liopeltis rappii*, we also generated molecular data for the type species of the genus *Gongylosoma*, *i*.*e*., *G. baliodeira* FMNH 269021, in addition to *G. scriptum*, *G. longicauda* (Peters, 1871) NHMW 26961:1 and *Liopeltis calamaria* (Günther, 1858). Genomic DNA was isolated from the preserved tissues of the specimens using QIAGEN DNeasy kits, following protocols directed by the manufacturer. Molecular methods largely follow Mirza et al.^22^ and Mirza and Patel^26^. A fragment of the mitochondrial cytochrome b (*cyt* b), *16 S* rRNA and nuclear Oocyte Maturation Factor mos (*c-mos*), neurotrophin-3 (*nt3*), and recombination-activating protein 1 (*rag-1*) genes were amplified using primers used by Pyron et al.^27^ and Mirza et al.^21^. A 22.4 µl reaction was set for a bi-directional Polymerase Chain Reaction (PCR), containing 10 µl of Thermo Scientific DreamTaq PCR Master Mix, 10 µl of molecular grade water, 0.2 µl of each 10 µM primer and 2 µl template DNA, carried out with an Applied Biosystems ProFlex PCR System. Thermo-cycle profile used for amplification were as follows: 95 °C for 3 min, (denaturation temperature 95 °C for 30 s, annealing temperature 48 °C for 45 s for *cyt* b & 16 S and 55 °C for *c-mos*, elongation temperature 72 °C for 1 min) × 36 cycles, 72 °C for 10 min, hold at 4 °C. PCR product was cleaned using QIAquick PCR Purification Kit and sequenced with an Applied Biosystems 3730 DNA Analyzer. Besides this, sequences analyzed by Zaher et al.^3^ of Colubridae available on GenBank^®^ were downloaded for inferring molecular phylogeny, and the sequences were concatenated using SequenceMatrix^28^. The first dataset comprised three nuclear genes, *c-mos*, *nt3*, and *rag1*, which was subjected to Maximum Likelihood (ML) phylogeny to elucidate the placement of *Liopeltis rappii*. Based on the results of the molecular phylogeny based on three nuclear genes (Supporting Figure S6), we assembled a second dataset of concatenated mitochondrial (*16 S*, *cyt b*, *nd4*) and nuclear (*c-mos* and *nt3*) genes, and more sequences were added to the dataset, especially mitochondrial sequences to populate the tree with more species. The sequences of *rag-1* were removed from the analysis as data for only a few species was available. Taxa for molecular phylogenetics were selected based on the tree topologies recovered by Zaher et al.^3^. Sequences were aligned in MegaX^29^ using ClustalW^30^ with default settings, and the sequence substitution model was selected with ModelFinder^31^. The aligned dataset was subject to phylogenetic analysis on the IQ-TREE (http://iqtree.cibiv.univie.ac.at/) online portal^32^. The analysis was implemented with a partition model for the concatenated dataset. The analysis was run with an ultrafast bootstrap option for 1000 iterations to assess clade support. Uncorrected pairwise *p*-distance (% sequence divergence) was calculated in MegaX with pairwise deletions of missing data and gaps. Sequences used in the phylogenetic analysis are listed in Supplementary Table S2, and sequence evolution model for individual analysis is in Supplementary Table S3.

## Results

### Molecular data

**Fig. 1:**
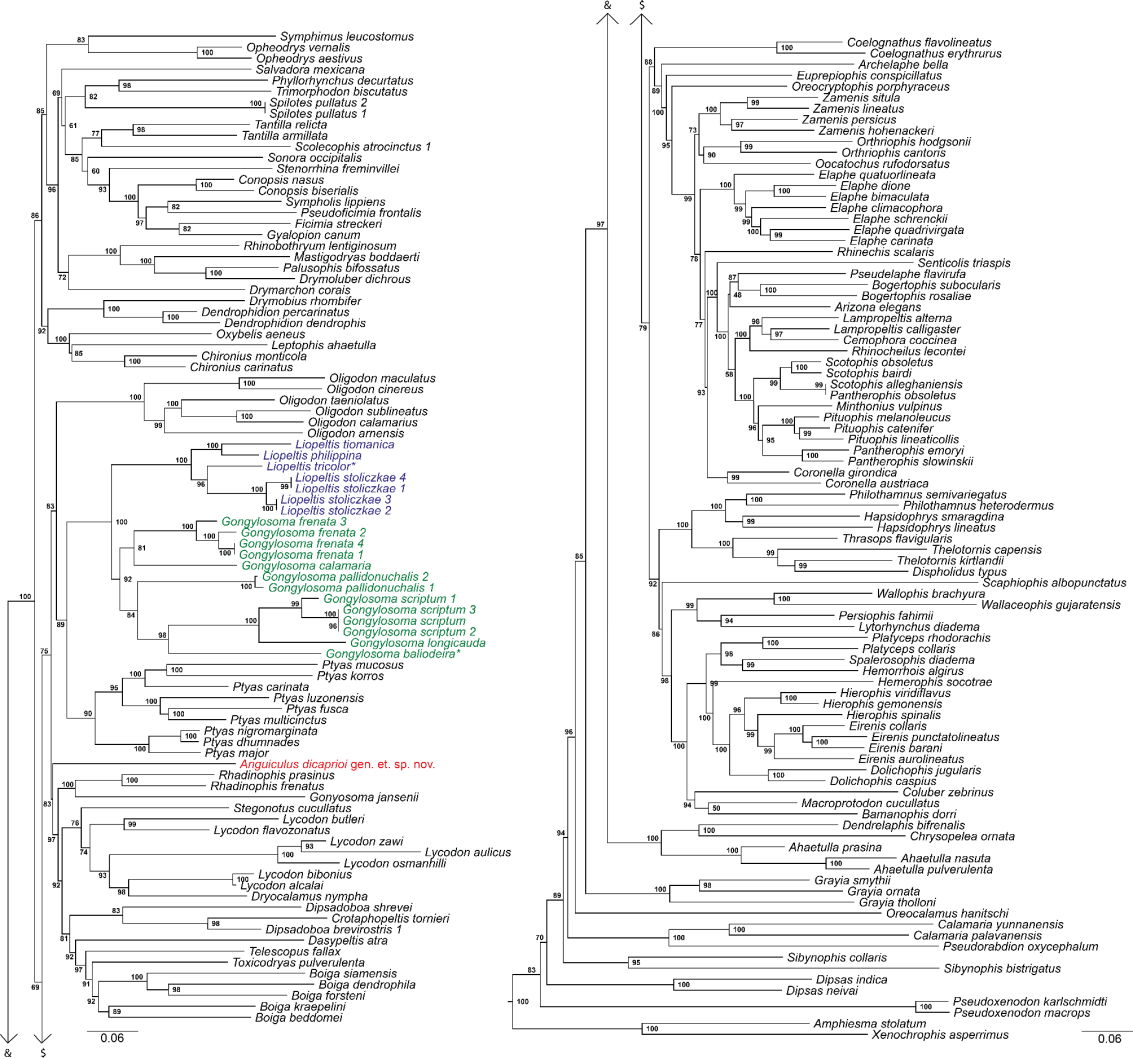
Maximum Likelihood phylogeny inferred for selected members of Colubrinae based 3647 bp of concatenated mitochondrial (*16 S*, *cyt b*, *nd4*) and nuclear (*c-mos* and *nt3*) gene showing phylogenetic relationship of *Anguiculus ***gen. nov.**, *Gongylosoma* and *Liopeltis*. For the complete tree, see supplementary Fig. S7.

ML phylogeny based on 2525 bp of nuclear genes (*c-mos*, *nt3*, and *rag-1*) recovered *Liopeltis rappii*, the genera *Liopeltis*, and *Gongylosoma* as members of the colubrid subfamily Colubrinae (Supplementary Figure S6). Based on the results from the nuclear dataset, a dataset comprised of 3647 bp of concatenated mitochondrial (*16 S*, cyt *b*, *nd4*) and nuclear (*c-mos* and *nt3*) genes recovered similar results wherein the population of ‘*Liopeltis rappii*’ from Himachal Pradesh as a member of the subfamily Colubrinae. It was found to be a member of the clade containing the genera *Boiga* Fitzinger, 1826, *Dasypeltis* Wagler, 1830, *Dipsadoboa* Günther, 1858, *Gongylosoma*, *Gonyosoma* Fitzinger, 1843, *Liopeltis*, *Lycodon* Boie *in* Fitzinger, 1826, *Oligodon* Boie *in* Fitzinger, 1826, *Ptyas* Fitzinger, 1843, *Rhadinophis* Vogt, 1922, *Stegonotus* Duméril et al., 1854, *Telescopus* Wagler, 1830 and *Toxicodryas* Hallowell, 1857 (Fig. 1). The species was not embedded within the genera *Liopeltis* nor *Gongylosoma*, furthermore, certain species of *Liopeltis*, namely, *L. frenata* and *L. pallidonuchalis* were embedded within the *Gongylosoma* clade, rendering *Liopeltis* paraphyletic (Fig. 1). Within Colubrinae, ‘*L. rappii’* from Himachal Pradesh was sister to a clade containing *Boiga*,* Dasypeltis*,* Dipsadoboa*,* Gonyosoma*, *Lycodon*, *Rhadinophis*, *Stegonotus*, *Telescopus* and *Toxicodryas* with moderate support (ML bootstrap 83). Phylogenetic relationships within the genera *Liopeltis sensus stricto* and *Gongylosoma sensus stricto* are well resolved with moderate to high support (ML bootstrap > 84). The species *L. calamaria*, *L. frenata*, and *L. pallidonuchalis* were recovered sister to the clade containing *G. baliodeira* and other species of the genus *Gongylosoma* and not *Liopeltis.* This finding necessitates the transfer of these three species (*L. calamaria*, *L. frenata*, and *L. pallidonuchalis*) to the genus *Gongylosoma*.

### Morphology data

**Fig. 2:**
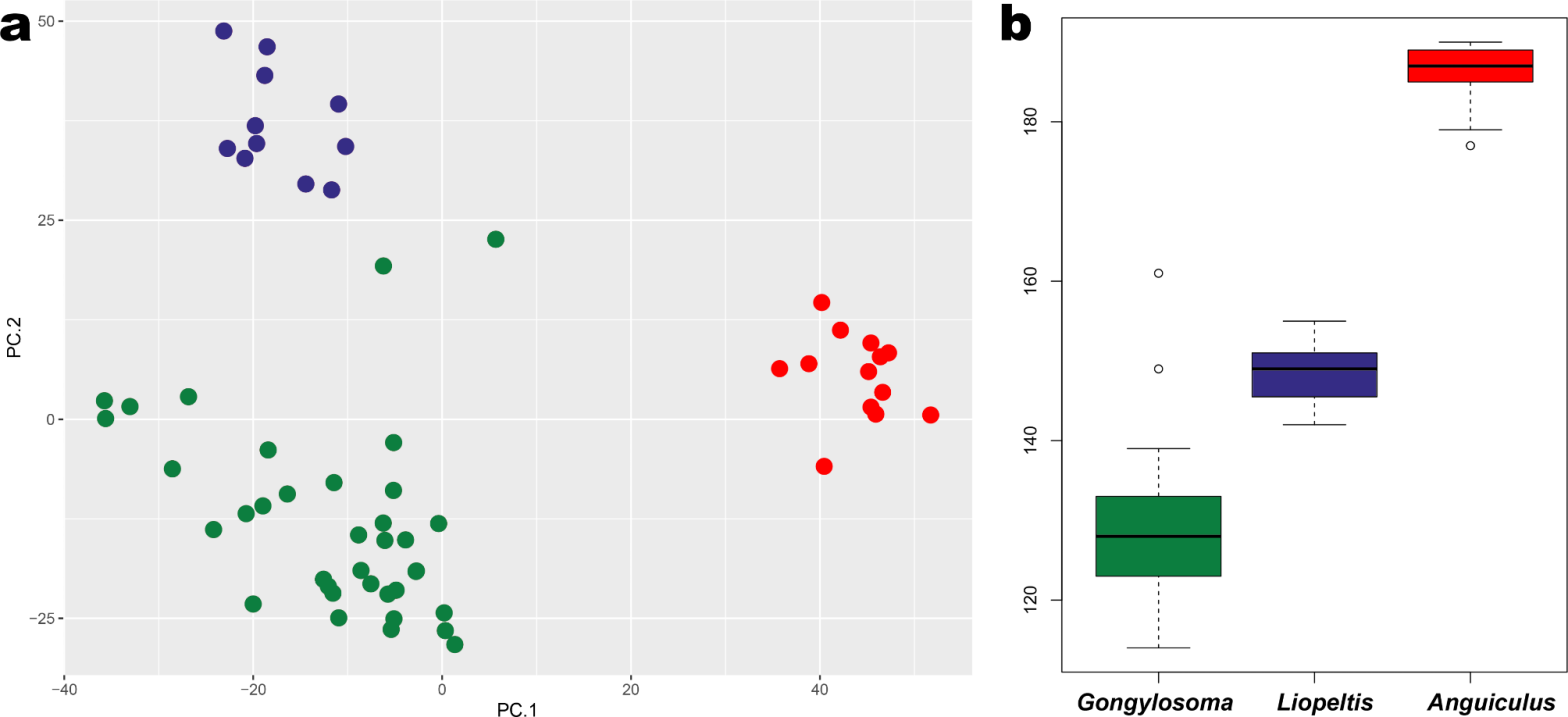
Principal Component Analysis plot (**a**) showing segregation of the three genera *Anguiculus ***gen. nov.** (red), *Gongylosoma* (green**)**, and *Liopeltis* (blue) in PC1 + PC2; (**b**) boxplot for the three genera showing the range of ventral scales in members of the three genera.

Small-sized snakes with smooth 15 DSR throughout, lacking apical pits, broad collar, sub-equal maxillary teeth (no teeth enlargement), loreal present and nostril laterally oriented between two nasals, eye with a rounded pupil, head sparsely distinct from neck and hemipenis extending to the 7th subcaudal, less than half of the organ calyculate; cusps are smaller at its distal portion gradually increasing towards the spinose base; numerous subequal spines except for two large ones at the base; the organ lacks fold which distinguishes *Liopeltis rappii sensu lato* from most Asian members of the subfamily Colubrinae, except for the genera *Liopeltis* and *Gongylosoma*. *Anguiculus ***gen. nov.** separates well from the genera *Liopeltis* and *Gongylosoma* in a PCA where PC1 + PC2 explain 58 + 41% of the variance observed with maximum loadings for ventral and subcaudal scales (Fig. 2a, Table S1). The new genus *Anguiculus ***gen. nov.** has a non-overlapping range of ventral scales with members of the genera *Liopeltis* and *Gongylosoma* (Fig. 2b). See comparison section for a detailed comparison with *Liopeltis* and *Gongylosoma*. Scalation data, skull osteology, and distribution support two diagnosable populations of *Anguiculus ***gen. nov.** The population from eastern Himalayas is here treated as *Anguiculus rappii sensu stricto* as the type locality is Sikkim, and the population from central and western Himalayas is described as a new species (see Systematics for details).

### Nomenclature

In order to stabilize the taxonomy, we redefine the genera *Liopeltis* and *Gongylosoma* based on the results from the molecular phylogeny and consider both genera as valid. To do so, two species *L. frenata* and *L. pallidonuchalis* are here transferred to the genus *Gongylosoma* as they are embedded within the *Gongylosoma* clade including the type species of the genus, *G. baliodeira*. A new genus is proposed to accommodate *L. rappii* and a new related species.

### Systematics

***Anguiculus*** **gen. nov.**

urn: lsid: zoobank.org: act:429639F9-1D88-4C76-9ECA-37EF90CE2A83.

Figures 3, 4, 5, 6, 7, 8, 9, 10 and 11; Tables 1, 2 and 3.

**Fig. 3:**
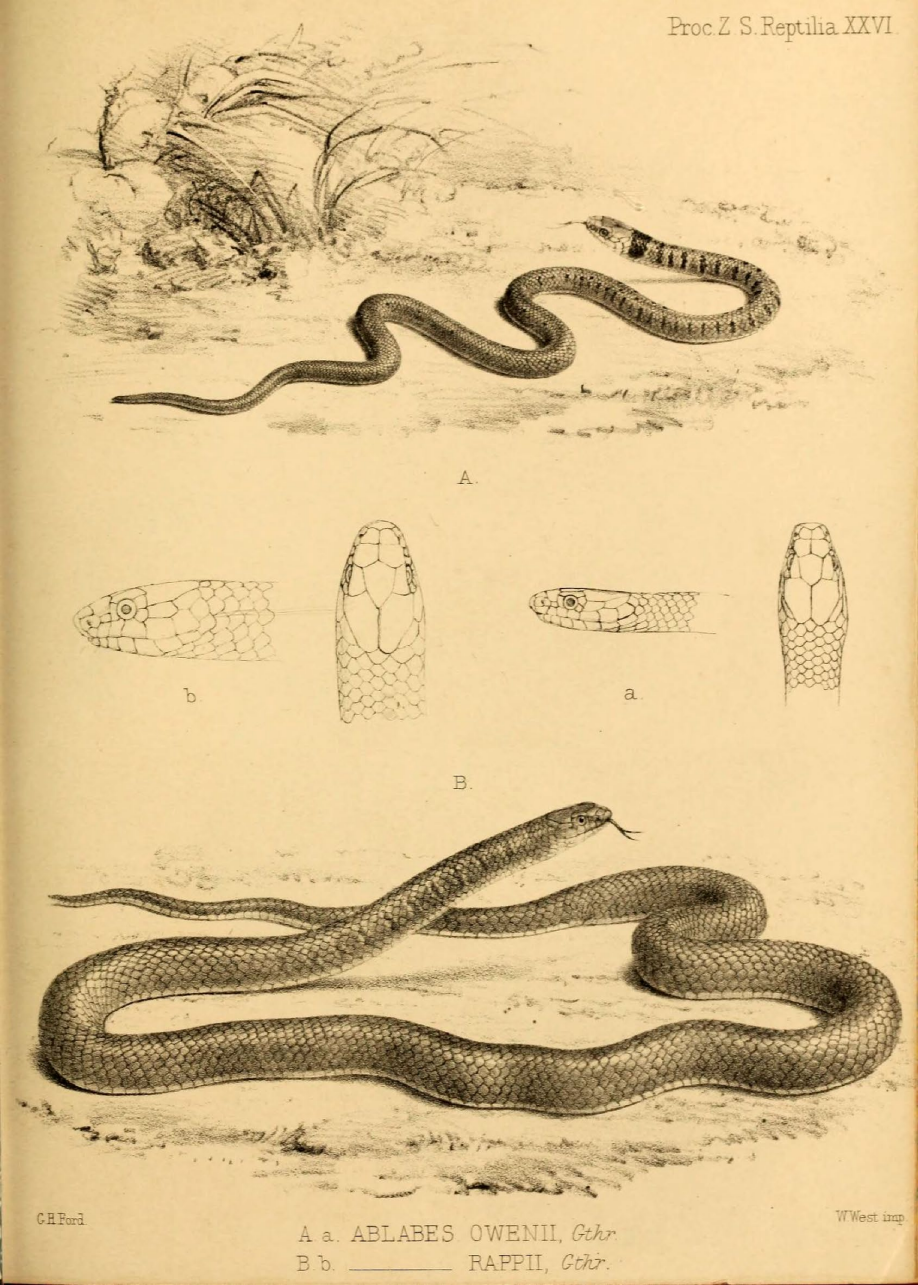
Plate contacting the original sketches of *Ablabes owenii* (= *Liopeltis rappii*) and *Ablabes rappii* (= *Liopeltis rappii*) from Günther (1860).

**Fig. 4:**
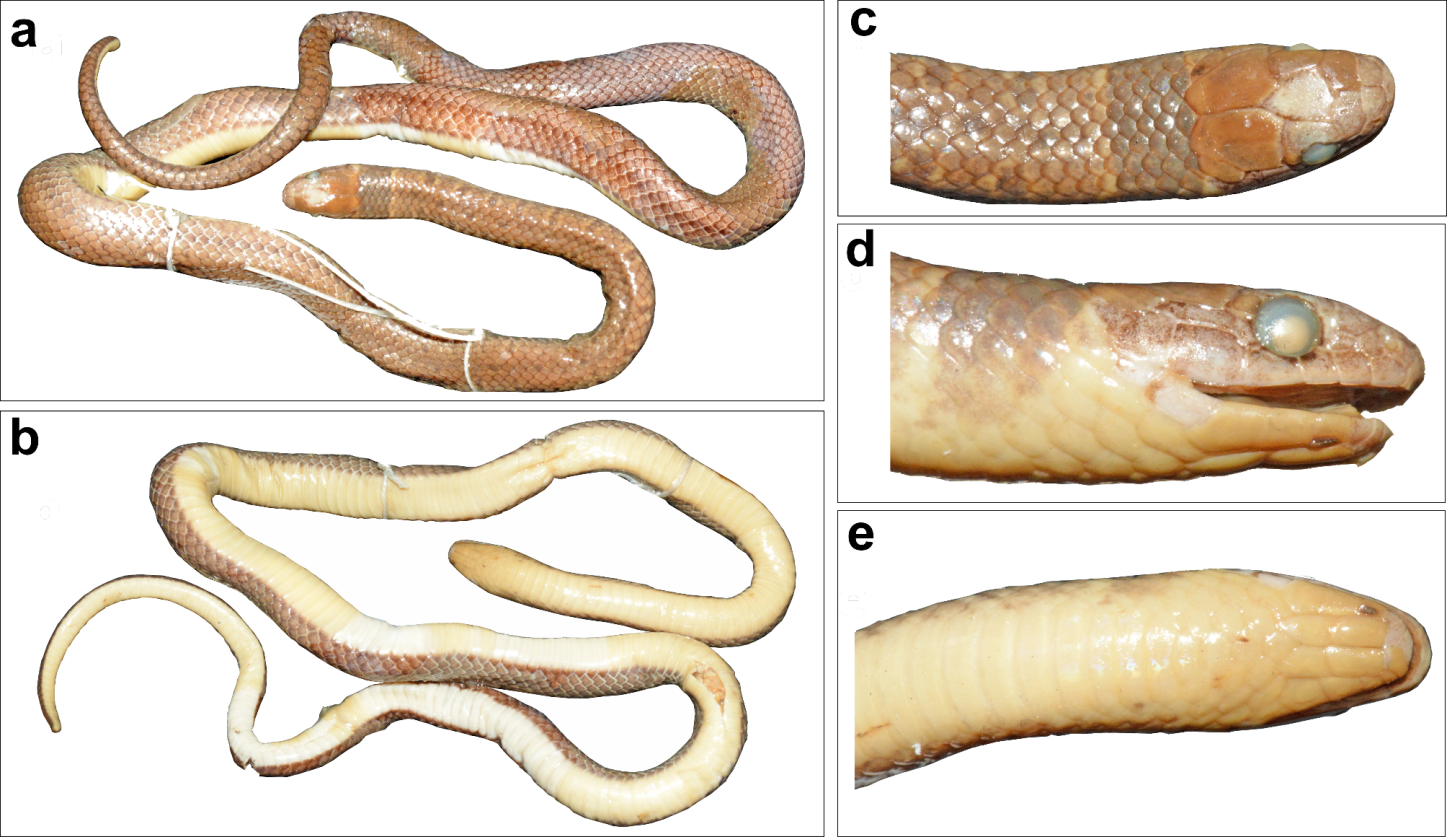
Holotype NHMUK 1946.1.5.61 of *Anguiculus rappii ***comb. nov.**, (**a**) dorsal view, (**b**) ventral view, (**c**) head dorsal view, (**d**) head lateral view, (**e**) head ventral view.

**Fig. 5:**
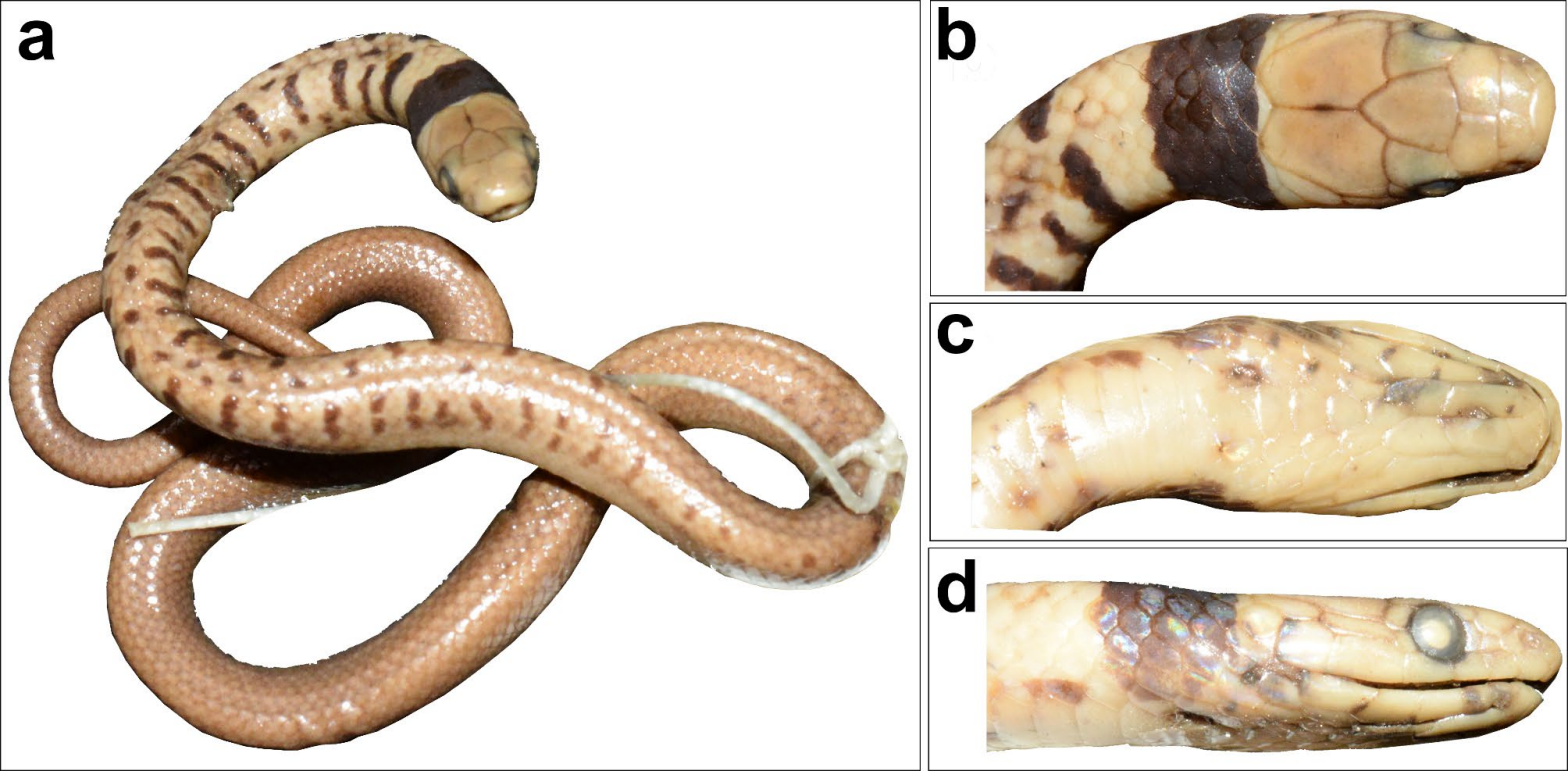
Holotype NHMUK 1946.1.5.56 of *Ablabes owenii* (= *Anguiculus rappii ***comb. nov.**), (**a**) dorsal view, (**b**) head dorsal view, (**c**) head ventral view, (**d**) head lateral view.

**Fig. 6:**
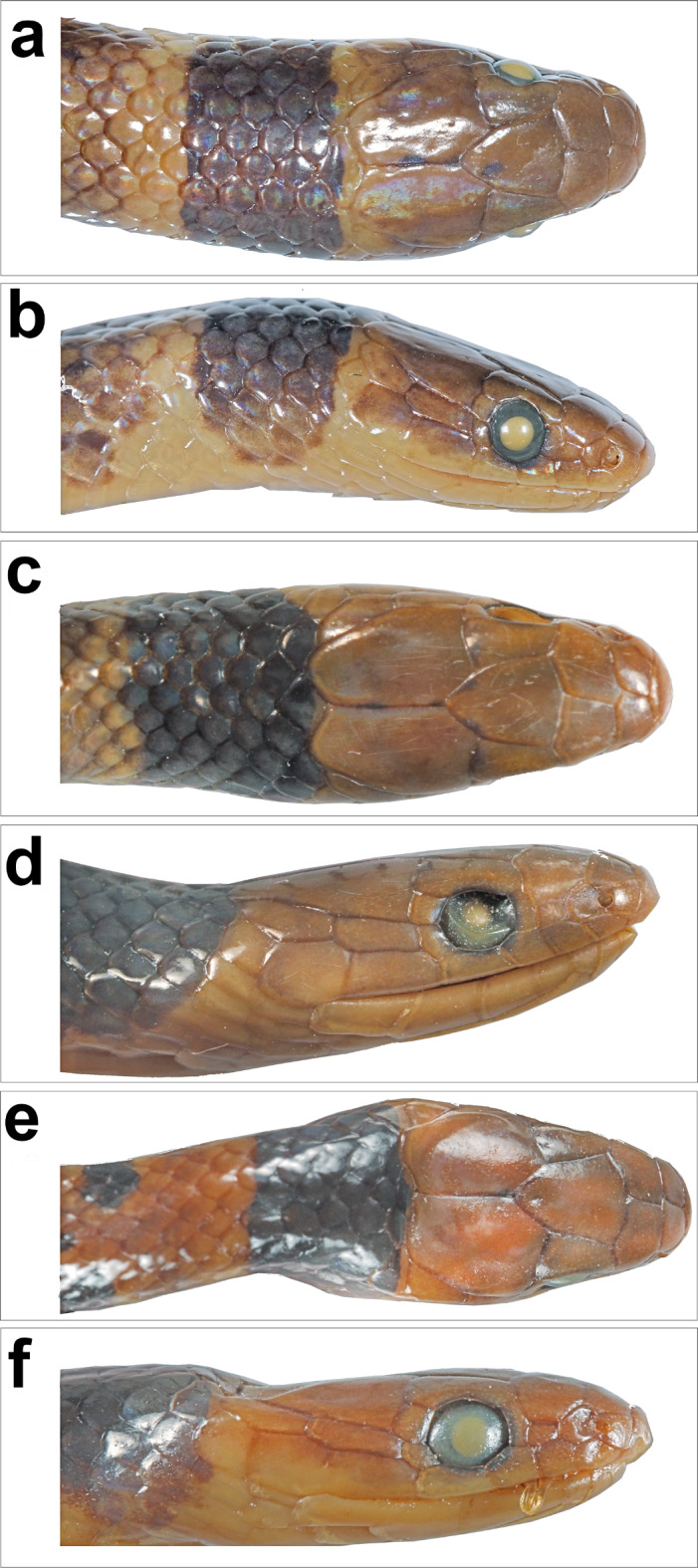
*Anguiculus rappii ***comb. nov.** showing head scalation, ‘a, c, e’ dorsal view and ‘b, d, f’ lateral view; (**a**,**b**) BNHS 744, (**c**,**d**) BNHS 747, (**e**,**f**) BNHS 750.

**Fig. 7:**
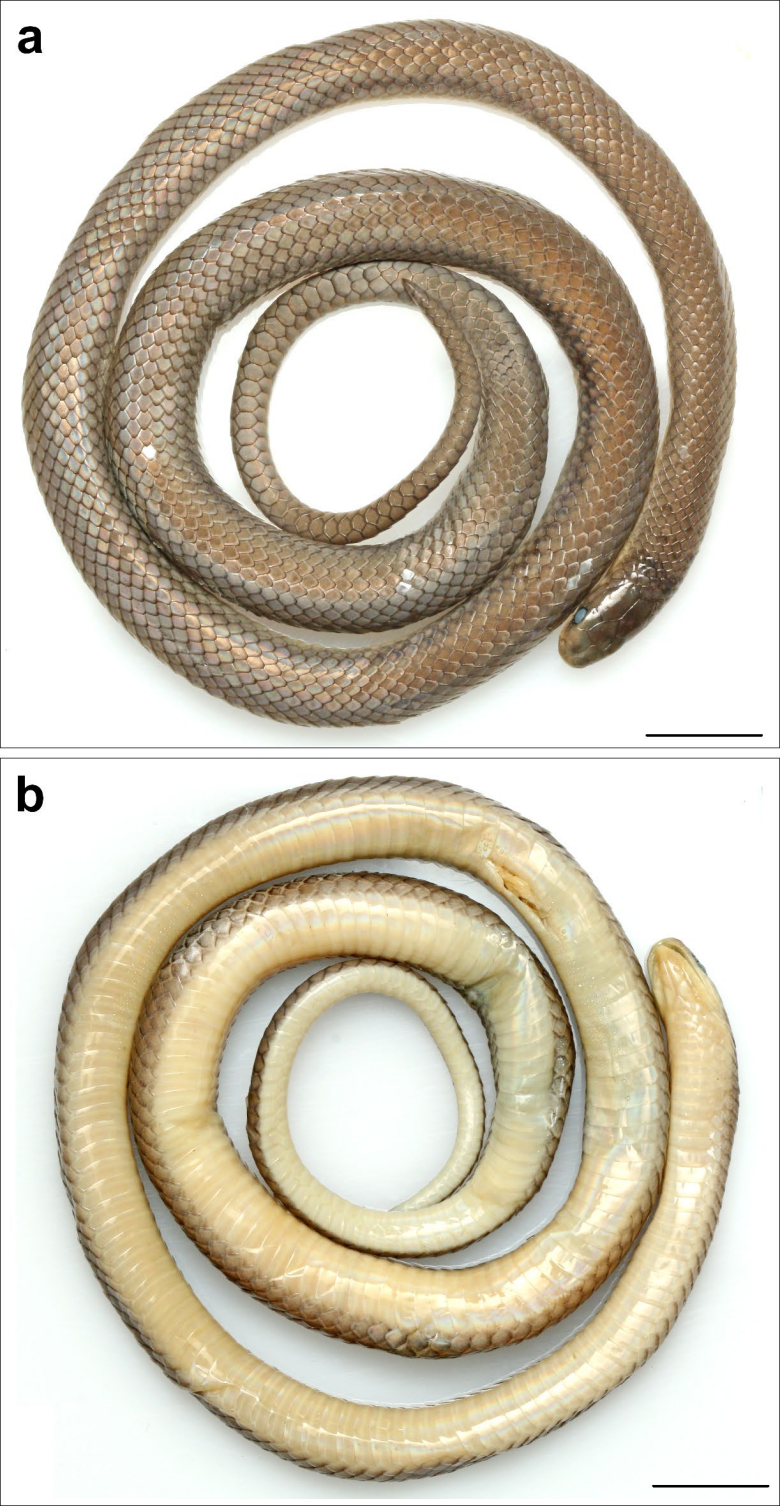
*Anguiculus dicaprioi ***gen. et. sp. nov.** holotype female NCBS NRC-AA-0013, (**a**) dorsal view, (**b**) ventral view. Scale bar 10 mm.

**Fig. 8:**
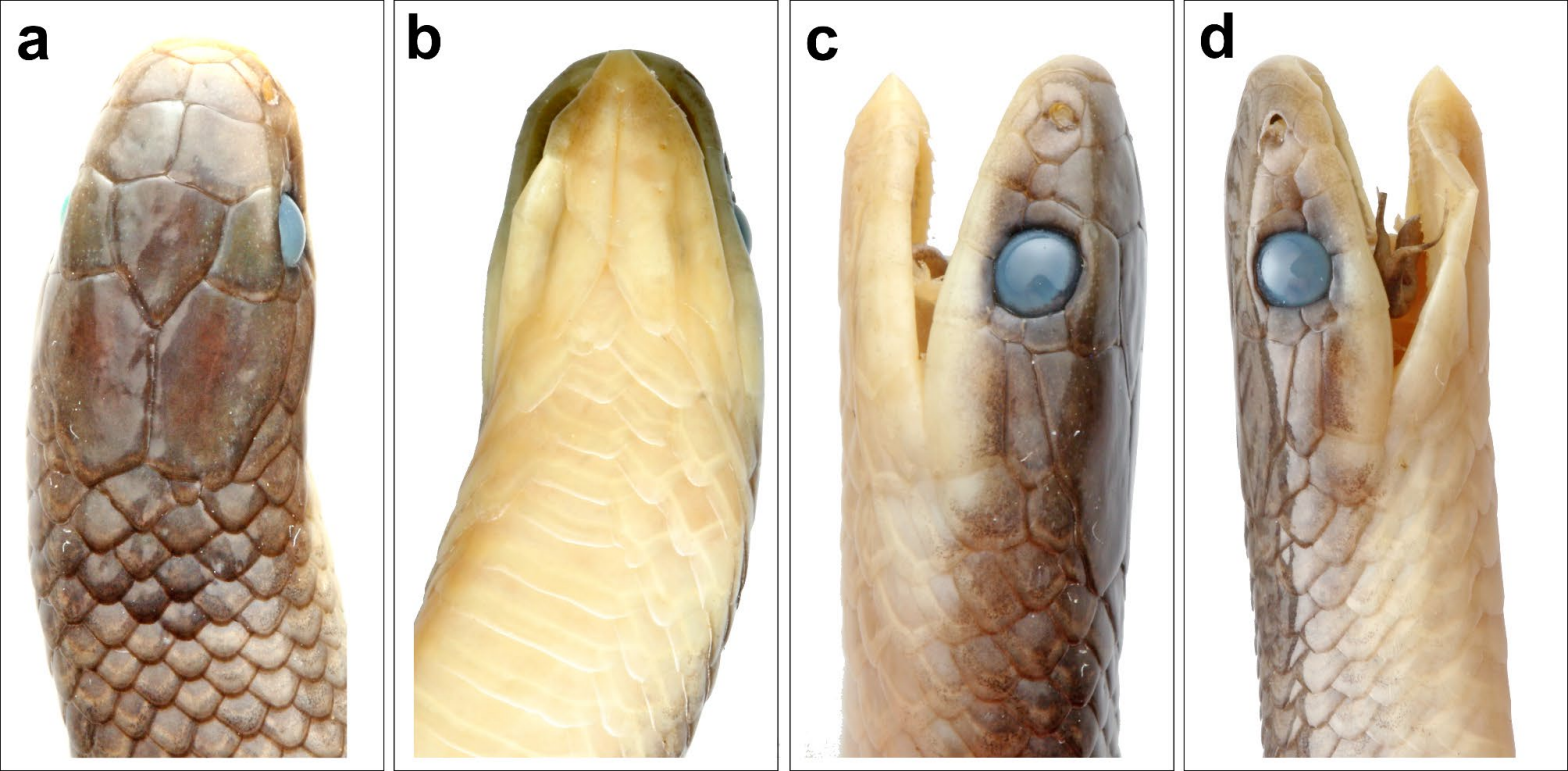
*Anguiculus dicaprioi ***gen. et. sp. nov.** holotype female NCBS NRC-AA-0013 showing head, (**a**) dorsal view, (**b**) ventral view, (**c**) left lateral view, (**d**) right lateral view.

**Fig. 9:**
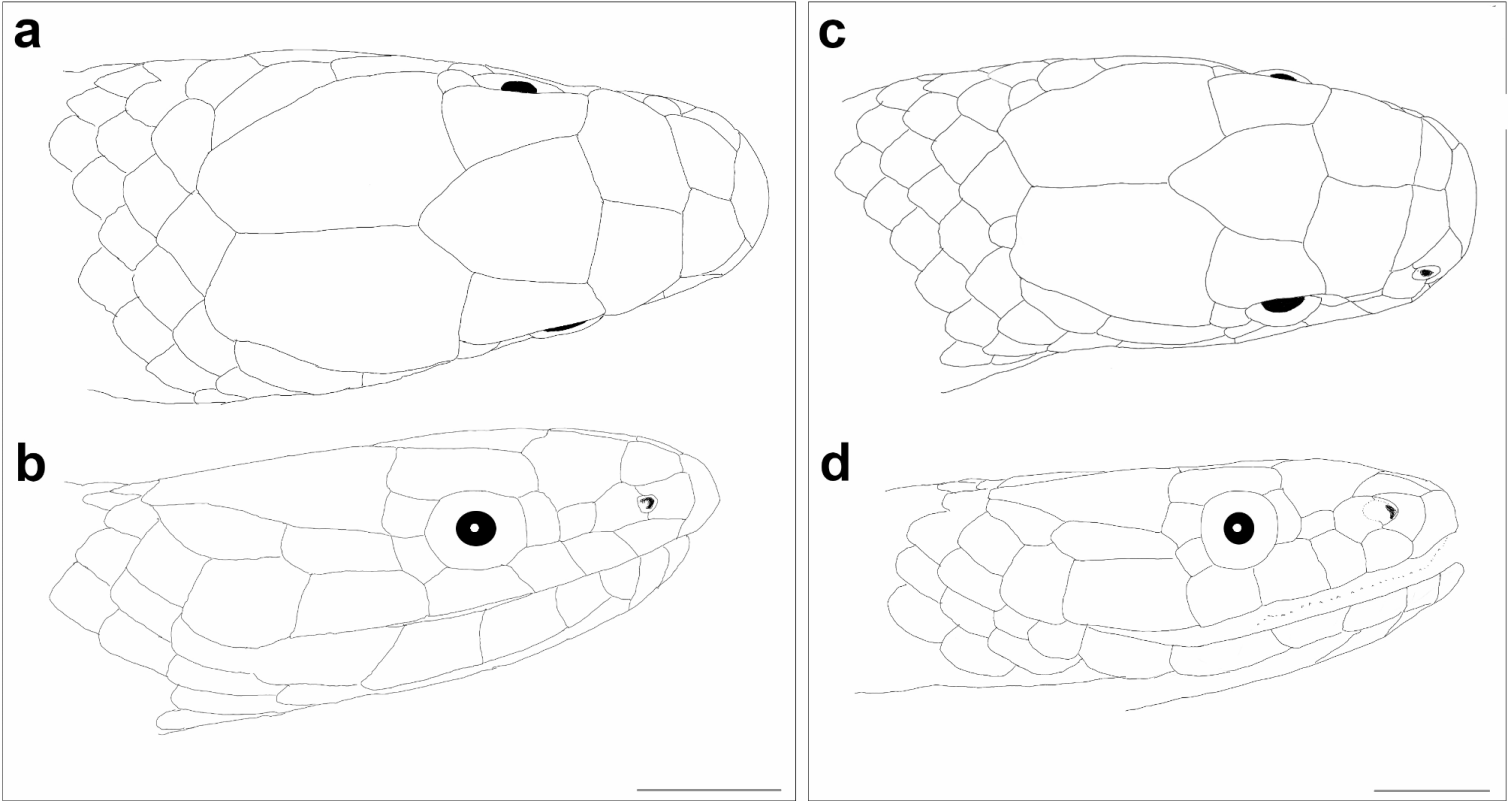
Schematic representation of head dorsal (**a**,**c**) and lateral (**b**,**d**) scalation of *Anguiculus ***gen. nov.**, (**a**,**b**) *Anguiculus rappii ***comb. nov.** and (**c**,**d**) *Anguiculus dicaprioi *sp. nov. Scale bar 2 mm.

**Fig. 10:**
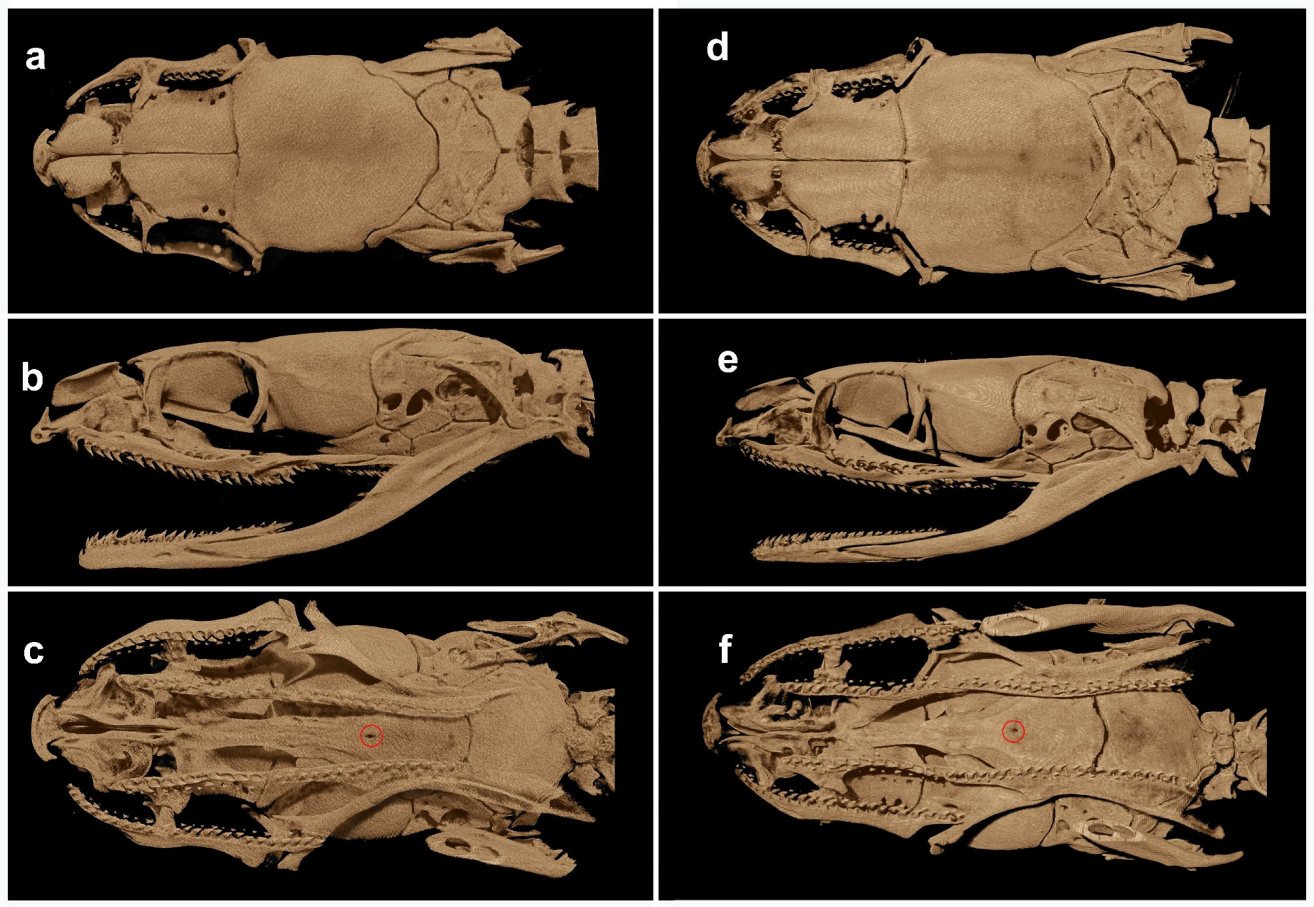
MicroCT images of the skull of *Anguiculus ***gen. nov.**, (**a**–**c**) *Anguiculus dicaprioi ***sp. nov.** NCBS NRC-AA-0013 & (**d**–**f**) *Anguiculus rappii ***comb. nov.** BNHS 743; (**a**, **d**) dorsal view, (**b**, **e**) lateral view, (**c**, **f**) ventral view.

**Fig. 11:**
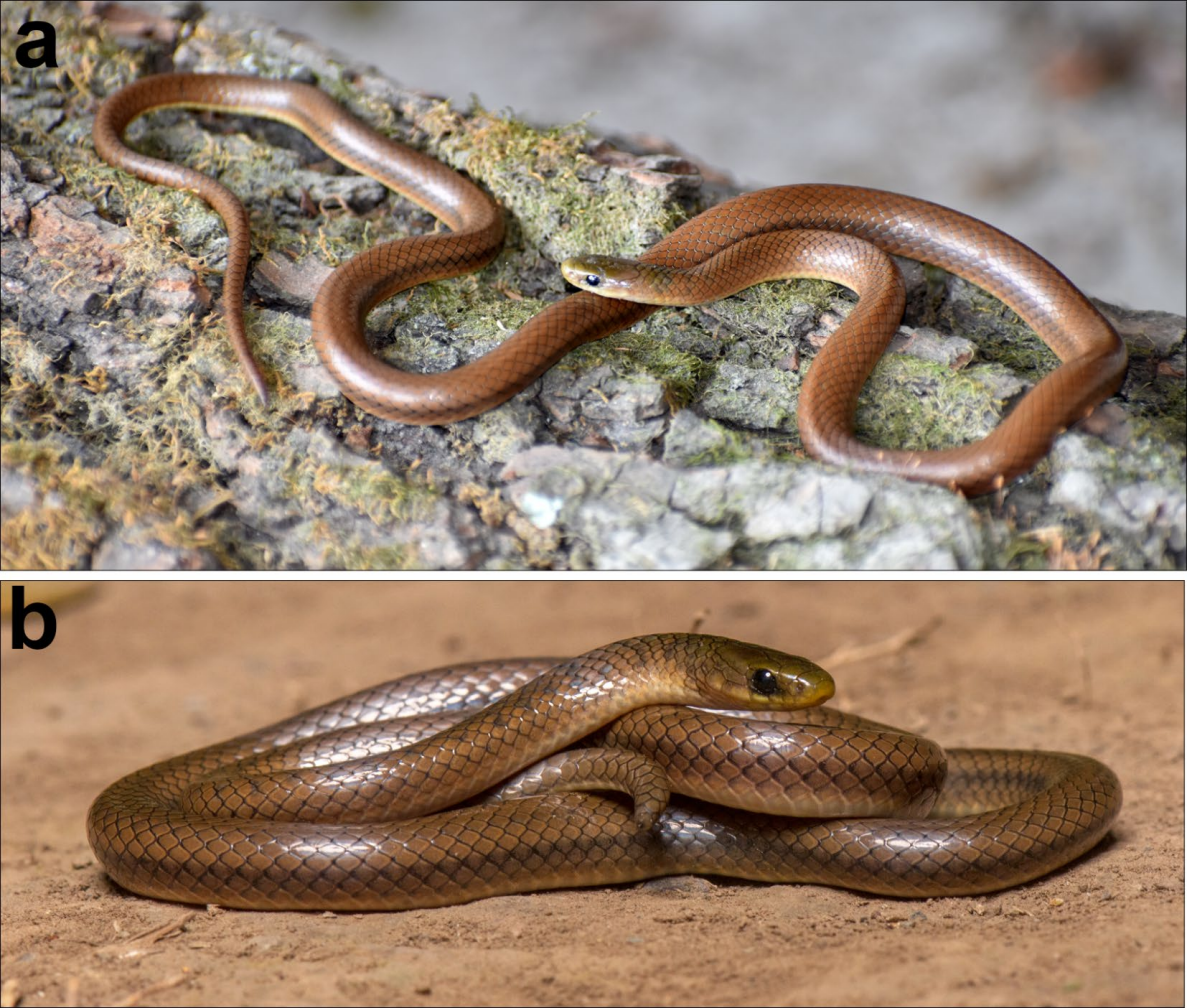
*Anguiculus dicaprioi ***gen. et. sp. nov.** in life, (**a**) holotype female NCBS NRC-AA-0013, Photo by Virender Bharadwaj; (**b**) uncollected individual from Nainital, Uttarakhand. Photo by Vipul Ramanuj.

**Table 1:** Morphometric and meristic data for *Anguiculus rappii ***gen. et comb. nov.**

Specimen no.	NHMUK 1946.1.5.61	NHMUK 1946.1.5.56	BNHS 743	BNHS 744	BNHS 745	BNHS 746	BNHS 747	BNHS 748	BNHS 751	ZMB 10325
Type status	Holotype (*Ablabes rappii)*	Holotype (*Ablabes owenii*)	–	–	–	–	–	–	–	–
Locality	Sikkim	Sikkim	Tindharia, Darjeeling	Tindharia, Darjeeling	Tindharia, Darjeeling	Tindharia, Darjeeling	Tindharia, Darjeeling	Tindharia, Darjeeling	Mishmi Hills	Darjeeling
TL	410	184.5	383	395	384*	290*	339	347	431	390
SVL	323	150	303	310	336	279	263	261	327	296
TaL	87	34.5	80	85	48*	11*	76	86	104	94
DSR	15:15:15	15:15:15	15:15:15	15:15:15	15:15:15	15:15:15	15:15:15	15:15:15	15:15:15	15:15:15
VS	185	184	187	187	183	180	182	179	187	189
A	Divided	Divided	Divided	Divided	Divided	Divided	Divided	Divided	Divided	Divided
SC	61	59	65	62	29*	6*	69	70	75	68
SL	6/5	6/6	6/6	6/6	6/6	5/5	6/6	6/6	6/6	6/6
LS	1/1	1/1	1/1	1/1	1/1	1/1	1/1	1/1	1/1	1/1
IL	7/7	6/6	7/7	7/7	7/7	7/7	7/7	7/7	7/6	7/7
PreO	1/1	1/1	1/1	1/1	1/1	1/1	1/1	1/1	1/1	1/1
PostO	2/2	2/2	2/2	2/2	2/2	2/2	2/2	2/2	2/2	2/2
STP	5	5	-	3	-	-	4	-	4	5
T	1 + 1/1 + 1	1 + 1/1 + 1	1 + 1/1 + 1	1 + 1/1 + 1	1 + 1/1 + 1	1 + 1/1 + 1	1 + 1/1 + 1	1 + 1/1 + 1	1 + 1/1 + 1	1 + 1/1 + 1
Sex	Male	Juvenile	Male	Male	Male	Female	Male	Male	Male	Male

All measurements in ‘mm’, values indicated by ‘*’ are incomplete as the specimen in damaged.

**Table 2:** Morphometric and meristic data for *Anguiculus dicaprioi ***gen. et. sp. nov.**

Specimen no.	NCBS NRC AA-0013	BNHS 3670	NCBS NRC AA-0014	NHMW 26963:1	NHMW 26963:2	NHMW 26962:1	NHMW 26962:2	NHMW 26962:3	ZSI 8370	ZSI 15759	ZSI HARC	ZSI HARC/R317
Type status	Holotype	Paratype	Paratype	Paratype	–	–	–	–	–	–	–	–
Locality	Chamba, Himachal Pradesh	Chamba, Himachal Pradesh	Chamba, Himachal Pradesh	Simla	Kulu at Kategarh	Rangoon Valley	Rangoon Valley	Rangoon Valley	Shimla	Bhimtal	Chamba	Chamba
TL	405	370	518	572	449	420	487	378	540	394	-	557
SVL	330	294	403	455	353	313	381	289	430	314	-	443
TaL	75	76	115	117	96	107	106	89	110	80	-	114
D	15:15:15	15:15:15	15:15:15	15:15:15	15:15:15	15:15:15	15:15:15	15:15:15	15:15:15	15:15:15	15:15:15	15:15:15
VS	190	177	185	190	185	187	190	189	185	185	176	192
A	Divided	Divided	Divided	Divided	Divided	Divided	Divided	Divided	Divided	Divided	Divided	Divided
SC	57	57	60	64	61	64	66	66	62	62	65	63
SL	6/6	6/6	6/6	6/6	6/6	6/6	6/6	6/6	6/6	6/6	6/5	6/6
LS	1/1	1/1	1/1	1/1	1/1	1/1	1/1	1/1	1/1	1/1	1/1	1/1
IL	7/7	7/7	7/7	7/7	7/7	7/7	7/7	7/7	7/7	7/7	6/7	7/7
PreO	1/1	1/1	1/1	1/1	1/1	1/1	1/1	1/1	1/1	1/1	1/1	1/1
PostO	2/2	2/2	2/2	2/2	2/2	2/2	2/2	2/2	2/2	2/2	2/2	2/2
STP	6	6	6	8	7	7	7	7	6	6	7	6
T	1 + 1/1 + 1	1 + 1/1 + 1	1 + 1/1 + 1	1 + 1/1 + 1	1 + 1/1 + 1	1 + 1/1 + 1	1 + 1/1 + 1	1 + 1/1 + 1	1 + 1/1 + 1	1 + 1/1 + 1	1 + 1/1 + 1	1 + 1/1 + 1
Sex	Female	Female	Male	Male	Female	Male	Female	Female	Female	Male	Male	Male

All measurements in ‘mm’.

**Table 3:** Morphological comparison of the two species of the genus *Anguiculus* gen. nov.

	*Anguiculus rappii* gen. et. comb. nov. (*n* = 10)	*Anguiculus dicaprioi* gen. et. sp. nov. (*n* = 10)
SVL	279–336	294–430
TaL	76–104	75–117
TaL/TL	0.18–0.24	0.19–0.25
DSR	15:15:15	15:15:15
STP	3 to 5	6 to 8
V	179–189	176–192
SC	59–75	57–66
Palatine teeth	14	12
Pterygoid teeth	22–23	18–20

Type species: *Anguiculus dicaprioi ***gen. et. sp. nov.**

Species included: *Anguiculus rappii ***comb. nov.** (Günther, 1860), *Anguiculus dicaprioi ***sp. nov.**

Diagnosis: Small-sized snakes characterized by 15 smooth DSR throughout; head not distinct form neck in adults; internasal not fused with nasal; nasal divided bearing a laterally oriented nostril between the two; eye of moderate size (not large) in relation to the head; 177–192 ventral scales; 57–75 subcaudal scales; anal divided; prefrontal not in contact with supralabial, 6 supralabials (rarely 5), extending beyond the angle of the jaw; loreal present; 1 preocular, 2 postoculars and temporals 1 + 1. The supraoccipital is chevron-shaped. The maxilla bears 22–24 subequal functional teeth. The teeth are arranged in a continuous manner, lacking a diastema, and no enlargement of teeth is observed. The basisphenoid bears median foramen. Hemipenis unilobed, distal less than half of the organ calyculate or spinose; numerous subequal spines present except for two large ones at the base; the organ lacks folds.

Etymology: The generic epithet is a Latin masculine noun that refers to a ‘small snake’. The proposed nomen highlights the small size (SVL) of members of the new genus in relation to members of the family Colubridae. Suggested common English name ‘Himalayan snake’.

Skull features (Fig. 10): The skull of *Anguiculus ***gen. nov.** is complete and robust (Fig. 10). The skull in dorsal view is nearly twice as long as wide (Fig. 10a, b). The nasals are less calcified compared to other bones, and the connections with the other sets of bones is not clear, as the signal is lost. The premaxilla appears to be connected only to the septomaxilla and not in contact with the nasals. There is a wide space separating the anterior half of the nasals and the septomaxilla. The frontals are twice as long as wide and sub-hexagonal. The parietal is large and nearly as long as wide. The anterior border of the parietal, bordering the frontals, bears a slight inwards curvature, whereas the posterior border bears and deep outwards curvature. In lateral view, the frontal bears a distinct dorso-lateral ridge to which the curvatures of the preocular and postocular align (Fig. 10b, e). The parietal, prootic, and supraoccipital supported by the quadrate, form the robust part of the skull. The supraoccipital is chevron-shaped. The maxilla bears 22–24 subequal functional teeth. The teeth are arranged in a continuous manner, lacking a diastema, and no enlargement of teeth is observed. The teeth are present from the tip of the maxilla to the maxilla-ectopterygoid joint. The palatine is linearly aligned with a slight outward curve in *A. rappii ***comb. nov.** (the palatine diverges outward) with a slight inward curve in *A. dicaprioi ***sp. nov.**, bearing 12–14 subequal teeth. The palatine starts from the midline of the vomer and extends to the level of the maxilla-ectopterygoid joint. The pterygoid bears 22–24 functional teeth throughout its inner margin. The ectopterygoid is slender at the base and gradually widens towards the distal end, which forms a deep fork. The inner arm of the form is slender and terminates into a slender horn, whereas the outer arm is broad. The ectopterygoid process of the maxilla meets the ectopterygoid at its fork (Fig. 10f). The basisphenoid bears median foramen. The dentary bears 18–21 functional teeth; the ones of the posterior end are much smaller than the anterior ones.

Comparison: The new genus shows genetic and morphological characters that overlap with genera related to Asian rat snakes and trinket snakes. Therefore, the morphological comparison of the new genus is restricted to selected genera based on phylogenetic affinity and morphological similarity occurring in the Indian subcontinent. Comparison is made for differing and non-overlapping characters: median basisphenoid foramen present (vs. absent in most colubrid genera in addition to *Coelognathus* Fitzinger, 1843, *Cyclophiops* Boulenger, 1888, *Elaphe* Fitzinger in Wagler, 1833, *Euprepiophis* Fitzinger, 1843, *Gongylosoma*,* Gonyosoma*, *Liopeltis*, *Oligodon*,* Oreocryptophis* Utiger, Schätti, & Helfenberger, 2005, *Orthriophis* Utiger, Helfenberger, Schätti, Schmidt, Ruf & Ziswiler, 2002, ), maxillary teeth subequal in size, posterior teeth not enlarged (vs. posterior teeth enlarged in *Boiga* Fitzinger, 1826, *Lycodon* Boie *in* Fitzinger, 1826, *Ptyas*, *Oligodon*,), DSR 15 throughout the body, smooth (vs. DSR at midbody, usually 17 in *Lycodon*, 13–17 in *Gongylosoma*, 23 in *Wallaceophis* Mirza, Vyas, Patel, & Sanap, 2016 & *Wallophis* Werner, 1929, 19–27 in *Elaphe* & *Coelognathus*, 20–23 in *Orthriophis* & *Euprepiophis*), head scarcely distinct from neck in adults (vs. distinct in *Boiga*,* Coelognathus*, *Elaphe*,* Euprepiophis*, *Gongylosoma*, *Gonyosoma*, *Lycodon*, *Oreocryptophis*,* Orthriophis*, *Ptyas*), loreal present (vs. absent in *Archelaphe* Schulz, Böhme & Tillack, 2011).

The new genus is most similar to members of the genera *Liopeltis* and *Gongylosoma* and is here compared with these in greater detail: median basisphenoid foramen present (vs. absent in most colubrid genera in addition to *Liopeltis* and *Gongylosoma*), paired posterior basisphenoid foramen absent (vs. present in *Liopeltis* and *Gongylosoma*), head not distinct from neck (vs. head moderately distinct from neck in *Liopeltis*, head strongly distinct from neck in *Gongylosoma*), 177–192 ventrals (vs. 139–161 in *Liopeltis*), supralabials 6 and the 6th supralabial extending beyond the angle of the jaw (vs. 8–9 that do not extend beyond angle of the jaw in *Liopeltis*, supralabials 6–8 that do not extend beyond angle of the jaw in *Gongylosoma*), hemipenis with two large & stout spines on each side of sulcus spermaticus (vs. large spines absent in *Liopeltis* and *Gongylosoma*).

***Anguiculus rappii *comb. nov. (Günther**,** 1860)**

*Ablabes rappii* Günther 1860: 154; Boulenger 1890: 307; Wall 1909: 351; Stoliczka 1870: 107.

*Ablabes owenii* Günther 1860: 155.

*Liopeltis rappi* Wall 1924: 865; Smith 1943: 186; Das 1996: 57.

*Liopeltis rappii* Wallach et al. 2014: 385.

Figures 3, 4, 5 and 6; Table 1.

Holotype. NHMUK 1946.1.5.61, from Sikkim, India (Figs. 3 and 4).

Material Examined (*n* = 14). NHMUK 1946.1.5.56, from Sikkim, India (holotype of *Ablabes owenii* Figs. 3 and 5); BNHS 743–748, all from Tindharia, Darjeeling, West Bengal, India, collected by Major Frank Wall; BNHS 749, from Darjeeling, West Bengal, India collected by Oscar Lindyceri; BNHS 750, from Turjur Tea Estate, Darjeeling, West Bengal, India collected by Oscar Lindyceri; BNHS 751, from Mishmi Hills, Assam (now in Arunachal Pradesh), India collected by H. W. Wells; CAS 17244 from an unknown locality collected by Col. R. H. Beddome; male ZMB 10,325 from Darjeeling, West Bengal, India collected by Blanchard, female SMF 19320 and female ZSIK 7175 from Darjeeling, West Bengal, India.

Diagnosis. A small-sized snake characterized by 15 smooth DSR throughout, 179–189 ventral scales; 59–75 subcaudal scales; anal divided; temporals 1 + 1, posterior temporal as long as anterior one; 3–4 rarely 5 scales bordering parietal; dorsum brownish, a broad collar with small dark brown spots and transverse bars on the anterior quarter or third of the body; venter pale yellow. The palatine bears 14 subequal functional teeth. TaL/TL 0.18–0.24.

Comparison. See the comparison section for *Anguiculus dicaprioi ***sp. nov.**

Etymology. The specific epithet “*rappii*” (Günther 1860), is a patronym honouring Wilhelm Ludwig von Rapp, a professor in zoology at the University of Tübingen, Germany. Suggested common name ‘Rapp’s Himalayan snake’.

Description based on examined material. Body long and moderately thin, TL 184.5–431 mm (*n* = 9), SVL 150–336 mm (*n* = 9); tail robust, tapering and moderately long, TaL 34.5–104 mm (*n* = 7), Tal/TL ratio 0.21–0.25 in adults (*n* = 6); head short, comprising 2.35–2.45% (*n* = 3) of total length in adults, longer than wide (HL/HW ratio: 1.5–2.1 in adults), slightly distinct from neck in adults; eyes circular with round pupil; nostrils large; snout elongate, extending beyond the lower jaw, body circular.

Head scalation. Details of head scalation of some specimens are shown in Figs. 1, 2, 3 and 4. Head scalation includes 1 rostral, 2 internasals, 2 prefrontals, 2 supraoculars, 1 frontal, and 2 parietals from dorsal view (Figs. 4, 5, 6 and 9). Rostral large, slightly smaller than the width of the frontal, thick, in contact with first supralabial, nasal and internasals posteriorly, well visible from above, curved onto upper snout surface; internasals in contact, wider than long, separated by a median suture; prefrontals in contact, sub-pentagonal, slightly wider than long; frontal large, bell shaped, longer than wide, anterior portion wider than the posterior portion; supraoculars sub-rectangular, longer than wide, anterior narrower; parietals large, longer than wide, longer than frontal, suture between parietals as long as frontal, each parietal in contact with two or three dorsal scales posteriorly; nasal vertically divided, posterior part smaller, nostrils open in anterior part of nasal; one loreal, longer than high; one vertically elongate, rectangular preocular, not reaching the upper surface of head; two vertically elongate, rectangular post oculars; one anterior temporal, sub-rectangular, more than twice longer than wide, one posterior temporal, almost as long or slightly smaller than the anterior one; two pair of chin shields, anterior pair sub-rectangular, elongate, longer than wide, posterior pair sub-rectangular, smaller than the anterior one; six supralabials in most cases (five in BNHS 746 ), 3rd and 4th touch the eye, 5rd longest and 6th highest; seven infralabials in most cases (six on the left side in BNHS 751), 1st pair in contact, 1st− 5th contact with anterior chin shields, 5th largest, touching posterior chin shields.

Body scalation. Ventrals 179–189, feebly angulate laterally; anal divided; subcaudals 59–75, paired, terminal scute forming the tip of the tail. Dorsal scale rows (DSR) smooth, in 15:15:15 rows, lacking apical pits, vertebral scales similar to other dorsal scales in shape and size.

Hemipenial morphology (based on Smith^6^): Hemipenis less than half of the organ calyculate; cusps are smaller at its distal portion gradually increasing towards the spinose base; numerous subequal spines except for two large ones at the base; the organ lacks folds.

Skull of BNHS 743 (Fig. 5). The skull of *Anguiculus rappii ***comb. nov.** is similar to that of *Anguiculus dicaprioi ***gen. et. sp. nov.** The maxilla bears 24 subequal teeth throughout the bone lacking a diastema. The palatine is linearly aligned with a slight outward curve (the palatine diverges outward) bearing 14 subequal teeth. The palatine starts from the midline of the vomer and extends to the level of the maxilla-ectopterygoid joint. The pterygoid bears 22–23 functional teeth throughout its inner margin. The palatine-pterygoid arc on either side is nearly parallel to each other.

Coloration in preservative. Dorsal background in a shade of light brown with a broad (4–5) dorsal scales wide dark brown to black band on the nape (Figs. 4 and 5). The posterior border of the band bears a yellowish fringe, which diffuses into the dorsal brown colour. The anterior 1/3rd of the snake bears yellow-edged dark transverse bands which are not connected in the vertebral region and these bands diminish as they progress towards the posterior part of the snake, being entirely absent in the posterior half. The outer lateral edge of the ventral scales to the first four dorsal scale rows in a shade of dark brown prominent from the midbody to the vent, which appears as a broad lateral stripe. The ventral aspect lacks markings and is an off-white to yellowish colour. The bands on juveniles are more prominent than those in the adults (Figs. 3 and 4).

**Fig. 12:**
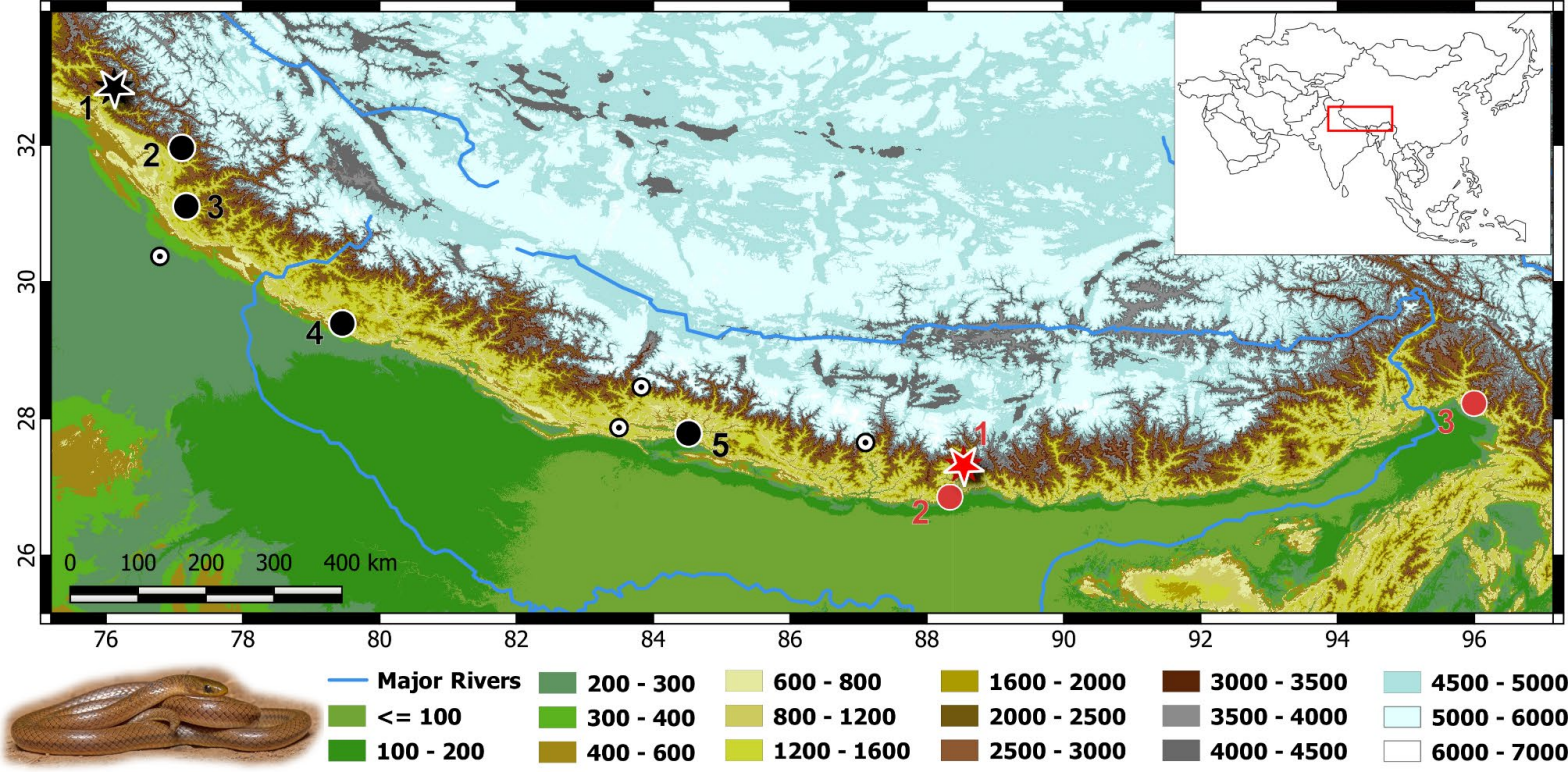
Map of the Himalayas and the Tibetan Plateau showing distribution of *Anguiculus ***gen. nov.**, *Anguiculus dicaprioi ***sp. nov.** marked in black, (1) Chamba, (2) Kullu, (3) Shimla, (4) Nainital, (5) Chitwan; *Anguiculus rappii ***comb. nov.** marked in red colour, (1) Sikkim, (2) Darjeeling, (3) Mishmi Hills. Star denotes type locality. Records denoted by a white circle with black dot require verification. Inset image *Anguiculus dicaprioi ***sp. nov.** Photo by Vipul Ramanuj.

Natural history and distribution (Fig. 12). Our observations coupled with published literature show that all the specimens originated from the Eastern Himalayas ranging from north Bengal to Arunachal Pradesh. The majority of the specimens came from two localities namely, Sikkim (India) and Tindharia (now in Darjeeling, West Bengal, India) except for one (BNHS 751), that originated from Mishmi Hills (Arunachal Pradesh, India). Records from Nepal require further verification as these records lack voucher specimens or images to confirm their identities (see Discussion).

Comments: The holotype of *Ablabes owenii* NHMUK 1946.1.5.56 (Figs. 3 and 5) was also described from Sikkim, India. The individual is a juvenile that bears a broad band on its nape and a few scattered spots on its anterior dorsum, that fades slightly in adult animals. The specimen matches all scalation characters that *A. rappii ***comb. nov.** exhibits and the difference in colouration can be attributed to ontogenetic variation. Thereby we retain it in the synonymy of *A. rappii ***comb. nov.**

***Anguiculus dicaprioi*** **gen. et. sp. nov.**

*Ablabes rappii* Stoliczka 1870: 107 (in part).

*Liopeltis rappi* Bhattarai et al. 2017: 89, Kuttalam et al. (2022): 85–88.

urn: lsid: zoobank.org: act:63A9F567-13F0-4C44-A840-CE9BB8E9DE6D.

Figures 7, 8, 9, 10 and 1111; Table 1.

Holotype: adult female, NCBS NRC-AA-0013 from near Thanei Kothi village, Churah Valley, Chamba District, Himachal Pradesh, India (32.835467, 76.119381, elevation 1864 m) collected by Virendar K. Bhardwaj on 15th June 2020.

Paratypes (*n* = 3): adult female BNHS 3670 and adult male NCBS NRC-AA-0014 from the same locality, collected on 22nd June 2020 and 29th June 2020, respectively by Virendar K. Bhardwaj; male NHMW 26963:1 from Shimla (Himachal Pradesh).

Additional referred material (*n* = 11): male MCZ R-4489 collected from Ambala, Haryana, India; female MCZ R-3137 & male MCZ R-3147 collected from Kooloo (Kullu) Valley, Himachal Pradesh, India; female ZSIK 8370 collected from Shimla, Himachal Pradesh; male ZSIK 15,759 collected from Bhimtal, Uttarakhand, India; female NHMW 26963:2 from Kullu, Himachal Pradesh; male NHMW 26962:1, two females NHMW 26962:2 & NHMW 26962:3 collected from Rangoon Valley by F. Stoliczka; male ZSI/HARC/R317 & juvenile ZSI/HARC/ without a number from Chamba, Himachal Pradesh cited by Kuttalam et al.^33^.

Diagnosis: A small-sized snake characterized by 15 smooth DSR in 15:15:15, 177–192 ventral scales; 57–66 subcaudal scales; anal divided; temporals 1 + 1, posterior temporal less than half the length of the anterior temporal; 6–8 scales bordering parietal; dorsum brownish, a diffused collar with small dark brown spots at the nape; venter white. The palatine bears 12 subequal functional teeth. TaL/TL 0.19–0.25.

Etymology. The specific epithet “*dicaprioi*” is a patronym honouring Leonardo DiCaprio, an American actor, film producer, and environmentalist who has been actively involved in creating awareness about global climate change, increased biodiversity loss, and human health issues through pollution. In addition to this, he has made funds available for field conservation activities and research. Suggested common name ‘DiCaprio’s Himalayan snake’.

Comparison: The new species, *Anguiculus dicaprioi ***gen. et. sp. nov.** differs from *Anguiculus rappii ***comb. nov.** as follows: posterior temporal less than half the length of the anterior temporal (posterior temporal as long as anterior one in *A. rappii ***comb. nov.**), 6–8 scales bordering parietal (3–5 in *A. rappii ***comb. nov.**), 57–66 subcaudal scales (59–75 in *A. rappii ***comb. nov.**), dorsum brownish, a diffused collar with small dark brown spots at the nape (dorsum brownish, a broad collar with small dark brown spots and transverse bars on the anterior quarter or third of the body in *A. rappii ***comb. nov.**), palatine with 12 functional teeth (vs. 14 in *A. rappii ***comb. nov.**). See Table 3 for a summary of morphological characters.

Description of female holotype NCBS NRC AA-0013:

The specimen is in good condition preserved in a coil with its head resting outside the coil. The specimen bears a small mid-ventral longitudinal incision (7–8 ventrals long) (Fig. 7a, b).

Head short, 6.4 mm comprising 1.6% of total length; high, 4 mm, with steeply domed snout in lateral view; upper jaw visible from ventral side. Head nearly as broad (5 mm) as the neck. Snout tapering to blunt, rounded tip in dorsal view (Fig. 8a). Rostral triangular, barely reaching top of the snout; wider (1.9 mm) than high (0.9 mm). Nostrils large, elliptical, between two sub equal nasal scales; nasal higher than long, posterior nasal deeply inserted between first and second supralabial. Paired internasals, wider (1.5 mm) than long (0.9 mm); smaller than prefrontals. Prefrontals, longer than wide (2 mm). Frontal bell shaped, 2.2 mm at the widest anterior border and much longer (3 mm). Parietals 3.9 mm long, 2.6 mm at its widest anterior. Temporals 1 + 1 on both sides, anterior one 2.5 mm long and 0.6 mm high, posterior one much smaller, 1.3 mm wide. Six scales, some of them slightly larger than adjacent dorsal scales, bordering parietals. Supraocular larger than preocular; preocular large, deeper than wide. Loreal twice as high (0.8 mm) as long (0.4 mm). Two postoculars, upper one larger. Eye circular, 1.5 mm diameter with a rounded pupil. Six supralabials, third and fourth in contact with orbit (Fig. 8c, d). First supralabial smallest and, apart from second supralabial, contacts only rostral and nasal. Second supralabial in contact with nasal, loreal, first + third supralabials and not in contact with the preocular. Third supralabial wider than the anterior ones, fourth subequal to third, fifth largest (long 2.4, high 1.2 mm). Sixth supralabial as long as high (1.2 mm) and extends beyond angle of jaw (Fig. 9c, d).

Mental short, broad, triangluar. Infralabials 7, anterior five infralabials short and thin, fifth onwards larger (Fig. 8b). First five infralabials in contact with the genials. Anterior genials more than twice as long (2.2 mm) as wide (0.8 mm); posterior genials 3 mm long and 1 mm wide and in contact (Fig. 8b).

Body rounded, not compressed laterally, ventral surface a little flattened. Dorsal scales in 15-15-15 rows. Ventral scales 190 (+ 3 preventrals) in number. Anal shield divided, slightly larger than last ventral scale. Subcaudals paired, 57 in number. Nine dorsal scale rows at base of tail reducing to two near the tip. Tail terminates in a sharp, tapering apical spine. Total length 405 mm, tail length 75 mm, tail/total length ratio 0.19.

Hemipenial morphology: the organ is fully everted and expanded (Fig. S3). Hemipenis short, unilobed, stout and unicalyculate; lobe extends for about half of the hemipenis; capitulum restricted to sulcate and dorsal surface. The organ is spinous almost throughout its length, with proximal end with few calyces dorsally; the spines are almost equal in size on the proximal half (truncus) of the hemipenis, gradually decreasing in size in the proximal end; sulcus spermaticus simple; the spine line on the either side of the sulcus spermaticus is weak; two large and stout spines, each on either side of the sulcus spermaticus present; hemipenial base is completely nude.

Colouration in preservative: Overall in a shade of brown dorsally, with vestigial dark brown band at the nape followed by a few disused dark brown spots. Ventrally whitish lacking markings or mottling. Lateral 4–5 rows of dorsal scales in a shade of greyish brown forming a longitudinal broad stripe from half the SVLup to the vent. The dorsum brown colouration barelyreaching half the depth of the supralabials and diffuses into the colouration of the venter. Colouration in life: The snout is olive, which gradually turns into a shade of brown with a tinge of orange from the nape that extends to the tail tip dorsally. A broad faint grey band at the neck. The dark brown to grey longitudinal stripes more distinct in life (Fig. 11).

Variation: The type series do not show any variation other than those noted in Table 1.

Skull of NCBS NRC-AA-0013 (Fig. 10a–c). The skull of *Anguiculus dicaprioi ***sp. nov.** is complete and robust, and similar to that of *A. rappii ***comb. nov.** in several regards (Fig. 10). The maxilla bears 25 subequal teeth throughout the bone lacking a diastema. The palatine is linearly aligned with a slight inward curve (the palatine converges inwards) bearing 12 subequal teeth. The pterygoid bears 18–20 functional teeth throughout its inner margin. The palatine- pterygoid arc on either side converges inwards.

Natural history and distribution: The holotype and the paratypes were collected from mud roads at the type locality. The individuals were seen basking and remained motionless until caught and made no attempts to bite. All the three type specimens were found in the month of June (2020). Uncollected individuals were observed in the months of July to September; however, no more individuals were seen in other months of the year. Sympatric serpents recorded from the area are *Lycodon bicolor* Nikolsky, 1903, *Oligodon russelius* Daudin, 1803, *Ptyas mucosa* Linnaeus, 1758, *Orthriophis hodgsonii* Günther, 1860, *Sinomicrurus* cf. *nigriventer* Wall, 1908, *Naja oxiana* Eichwald, 1831, *Daboia russelii* Shaw & Nodder, 1797 and *Gloydius chambensis* Kuttalam, Santra, Owens, Selvan, Mukherjee, Graham, Togridou, Bharti, Shi, Shanker & Malhotra, 2022 (Mirza et al.^34^). Nothing else is known about the biology of this species. Based on reliable reports, the species is currently known from the type locality, i.e. Chamba^33^, Kullu (MCZ R-3137 & R-3147) and Shimla in Himachal Pradesh, Nainital in Uttarakhand (Fig. 12) and Chitwan National Park in Nepal^19^.

***Gongylosoma *Fitzinger**,** 1843**.

Type species: *Gongylosoma baliodeira* (Boie, 1827).

Species included: *Gongylosoma baliodeira*, *Gongylosoma calamaria* (Günther, 1858) **comb. nov.**, *Gongylosoma frenata* (Günther, 1858) **comb. nov.**, *Gongylosoma longicauda*, *Gongylosoma mukutense* Grismer, Das & Leong, 2003, *Gongylosoma nicobariensis* (Stoliczka, 1870), *Gongylosoma pallidonuchalis* (Poyarkov, Nguyen & Vogel, 2019) **comb. nov.**, *Gongylosoma scriptum*.

Diagnosis: Small-sized snakes characterized by 13, 15 or 17 smooth DSR; head sparsely distinct form neck in adults; internasal not fused with nasal; nasal divided or fused with loreal, bearing a laterally oriented nostril between the two; eye of moderate size (not large) in relation to the head; 118–158 ventral scales; 58–125 subcaudal scales; anal divided; 6–8 supralabials, not extending beyond the angle of the jaw; one preocular, two postoculars and temporals 1 + 1 or 2 (1 + 2 in *G. nicobariensis*). The basisphenoid lacks median foramen. The maxilla bears 20–24 subequal functional teeth. The teeth are arranged in a continuous manner, lacking a diastema and no enlargement of teeth is observed. The basisphenoid lacks median foramen. Hemipenis unilobed or in some species, with weekly differentiated apical lobes; transverse or longitudinal folds present or absent; distal half with small papillae or nodules or with calyces, proximal half with slightly larger spines. See Table 4 for a summary of characters.

**Table 4:** Summary of diagnostic characters for the genera *Liopeltis*, *Gongylosoma* and *Anguiculus* gen. nov.

	*Liopeltis*	*Gongylosoma*	*Anguiculus* gen. nov.
DSR	15	13 or 15 or 17	15
V	139–161	118–158	177–192
SC	110–136	58–125	57–75
Head distinct from neck	Moderately	Strongly	Indistinct
Eye	Smaller in relation to the head	Larger in relation to the head	Larger in relation to the head
Suparalabials	8 to 9	6 to 8	5 or 6
Suparalabials exceeding the angle of the jaw	no	no	yes
Temporals	1 + 2	1 + 1 or 2	1 + 1
Maxillary teeth	28–31	20–24	22–24
median basisphenoid foramen	Absent	Absent	Present
Posterior paired basisphenoid foramen	Present	Present	Absent
TailL/TotalL	0.35–0.38	0.23–0.31	0.21–0.25
Hemipenis	Distal portion with large thorn-like spines	Proximal portion with larger spines; organ with or without folds	2 large & stout spines on each side of sulcus spermaticus; organ lacks folds

***Liopeltis *Fitzinger**,** 1843**.

Type species: *Liopeltis tricolor* (Schlegel, 1837).

Species included: *Liopeltis philippina* (Boettger, 1897), *Liopeltis stoliczkae* (Sclater, 1891), *Liopeltis tiomanica* Som, Grismer, Grismer, Wood, Quah, Brown, Diesmos, Weinell & Stuart, 2020, *Liopeltis tricolor* (Schlegel, 1837).

Diagnosis: Small-sized slender snakes characterized by 15 smooth DSR; head distinct form neck in adults; nasal undivided (may be fused with internasals) bearing a laterally oriented nostril; eye of moderate size (not large) in relation to the head; 139–161 ventral scales; 110–136 subcaudal scales; anal divided; prefrontal not in contact with supralabial, 8–9 supralabials, not extending beyond the angle of the jaw; loreal present; 1–2 preoculars, two postoculars and temporals 1 + 2. The maxilla bears 28–31 teeth. The teeth are arranged in a continuous manner, lacking a diastema, and no enlargement of teeth is observed. The basisphenoid lacks median foramen. See Table 4 for a summary of characters.

## Discussion and conclusion

Phylogenetic relationships across most colubrid genera have been well resolved^2–4,35^. The lack of molecular data of certain rare species is a major impediment in resolving the phylogenetic position of certain genera. The phylogenetic placement of *Anguiculus ***gen. nov.** is uncertain despite employing multiple markers to resolve its phylogenetic position. This uncertainty, largely may be attributed to the lack of molecular data for related taxa, especially *A. rappii ***comb. nov.**. The snake, *A. rappii ***comb. nov.** is extremely rare and there have been no reports of the species from the eastern parts of its range since Smith’s compilation in 1943^6^. However, our preliminary results show that the new genus is a member of the subfamily Colubrinae and is not embedded in the clades containing *Liopeltis* and *Gongylosoma*. *Anguiculus ***gen. nov.** bears a broad collar band, 15 DSR, nostril between nasals, and a median foramen on the basisphenoid. The presence of a basisphenoid median foramen in the two species of *Anguiculus ***gen. nov.** might serve as a diagnostic character. The distinction between the genera *Liopeltis* and *Gongylosoma* morphologically has been difficult in the past^8,9^; however, molecular data for the type species of the genus *Gongylosoma*, i.e. for *G. baliodeira*, made phylogenetic and morphological definition of the genera plausible.

The east Himalayan population of *Liopeltis rappii* is here referred to as *Anguiculus rappii ***comb. nov. ***sensu stricto* ranging from Darjeeling in West Bengal and Sikkim to the Mishmi hills in Arunachal Pradesh (Fig. 12). After examining a series of specimens of the former, we confirm that *Ablabes owenii* is indeed a synonym of *Anguiculus rappii ***comb. nov.** The population from central and western Himalayas^19,33^ is treated as a new species, *Anguiculus dicaprioi ***gen. et. sp. nov.**, that is at present known from Chamba, Kullu, Shimla in Himachal Pradesh, Nainital in Uttarakhand (India) and Chitwan National Park in Nepal. Other records from across Nepal require further verification, as these records were unpublished and lacked images or vouchers^19^. These records require proof to ascertain if they represent the new species (*Anguiculus dicaprioi ***gen. et. sp. nov.**) or *Anguiculus rappii ***comb. nov.**, especially those records from eastern Nepal. The three specimens NHMW 26962:1–3, were collected by F. Stoliczka from Rangoon Valley. It is quite unclear at this moment if these specimens were incorrectly labelled to refer to Yangon (Myanmar) or a locality in the western Himalayas that Stoliczka visited twice between 1864 and 1865^36^. Records of the species from Yangon (Myanmar) require confirmation as the species has not been reported from Myanmar^6,37^.

Snakes of the genera *Liopeltis*, *Gongylosoma*, and *Anguiculus ***gen. nov.** exhibit few diagnostic characters across congeners. Due to this high degree of morphological crypsis, several of these cryptic species have been merged under a single widespread taxon^8^ or were incorrectly identified^38^. Examples of morphologically cryptic species groups are *Gongylosoma calamaria ***comb. nov.**, *L. stoliczkae*, and *G. scriptum* evident from high genetic divergence across available sequences^17^ (Z. Mirza personal observation). An integrated approach corroborating morphology, molecular, and ecological data will have to be employed to dissociate these cryptic species.

Based on the current members of the *Liopeltis* and their distribution, it is likely that the ancestors of *Liopeltis* originated in Sundaland and dispersed westwards through the montane and lowland evergreen forests where their current distribution is restricted. Conversely, *Gongylosoma* is more widespread and occupies varied habitats, and the lack of molecular data of some species makes it difficult to postulate any biogeographic scenarios for this genus.

The Himalayas are a known biodiversity hotspot^39^, and the eastern front harbors higher diversity than its western counterpart^40^. However, the findings in the present work support the fact that the biota of the Western Himalayas is distinct^34,41,42^ and not merely a subset of the East Himalayan biota^40^. More work across taxa is necessary to document species, especially through an integrated taxonomic approach to unmask hidden diversity currently deprived of recognition and eventual conservation attention.

## Electronic supplementary material

Below is the link to the electronic supplementary material.

Supplementary Material 1

Supplementary Material 2

Supplementary Material 3

Supplementary Material 4

Supplementary Material 5

Supplementary Material 6

Supplementary Material 7

## Data Availability

All sequences generated in the present work have been submitted to the National Centre for Biotechnology Information and you may contact the corresponding author for any queries.
