# Supplementary material for: Phylogeny and systematics of the colubrid snake genera Liopeltis and Gongylosoma (Squamata: Colubridae) and description of a new Himalayan endemic genus and species
Source: Sci Rep. 2024 Oct 21;14:24743. doi: 10.1038/s41598-024-74271-1 (PMC11494134; doi:10.1038/s41598-024-74271-1)
Supplement: Supplementary file 1 — Supplementary Material 1 [file 41598_2024_74271_MOESM1_ESM.docx]

**Comparative material examined:**

*Gongylosoma baliodeirum*: males NMW 26970:3, Java; NMW 26974:1, Naga Padang, Padang; NMW 26974:2, Moara Terweh, Kalimantan; ZMH R11566, Borneo; NMW 26973:2, Lahat, Sumatra;NMW 26973:1, Deli Sumatra; NMW 26971:2, Java; NMW 26972:3, Padang; NMW 26972:6, Padang; RMNH 188, Sumatra; USNM 94826, Kao Soi Dao, Thailand, Trang; NMW 26972:5, Padang; NMW 26972:7, Padang; NMW 26972:2, Padang.

*Gongylosoma calamaria*: males NMW 26966:1, Ceylon; NMW 26966:2, Ceylon; NHM 1946.1.21.64, Ceylon; ZMB 10330, Darjeeling; female ZMB 10330, Darjeeling; ZMUC R-603579 Shervay hills; ZSIK 26943 Deormalai, Odisha.

*Gongylosoma frenatus*: males NHM 1946.1.1.72, Khasi Hills; BNHS 725–726, Assam; BNHS 727, Silumkaba, U. Burma; MZMU965 Mizoram, India.

*Gongylosoma longicaudum*: males NMW 26960:1, Nanga Padang; NMW 26961:1, Sumatra; NMW 26961:2, Nias; RMNH 4328, Kaja Taman, Sumatra; SMF 19326, Deli, Sumatra; SMF 43964, Sumatra; NMB 1638, Sumatra; females NMW 26960:2, Kalimantan; NMB 5127, Palembang, Sumatra.

*Gongylosoma nicobariensis*: ZSIK 7201 (holotype), Nocobars.

*Gongylosoma pallidonuchalis*: male ZMMU R-15682, Gia Lai Province, Vietnam; female ZFMK 83105, Bach Ma, Vietnam.

*Gongylosoma scriptum*: ZSIK 7207 (holotype), Martaban; males ZMB 50677, Khao Yai; MZMU 914, Mizoram, India; female MZMU 892, Mizoram, India; female ZMB 5286, East India.

*Liopeltis tricolor*: males RMNH 4036, Ind. Archip.; NHM 69.12.4.127, unknown locality; NMW 26968:1Penang; ZFMK 16691, Mt. Brinchang, Malaysia; NMW 26968:3, Moara Terweh, Kalimantan; NMW 26968:2 Padang, Sumatra; SMF 81197, Sumatra; NHM 1946.1.5.37, Java; NMW 26967:3, Java; ZMH 5229 old nr Buitenzorg, Java; females NMW 26967:2, Java; NMW 26967:1, Java; ZFMK 33533, Sumatra; NMW 26967:4, Java; RMNH 554, Banka; SMF 19317, West Borneo.

*Liopeltis stoliczkae*: male BNHS 728 Sittong Mungpoo, Darjeeling; ZSIK 3955 (syntype), Naga Hills; ZSIK 21905, Shillong, Meghalaya; MZMU965, MZMU1597 & MZMU1420, Mizoram.
