# Supplementary figures and images for: Phylogeny and systematics of the colubrid snake genera Liopeltis and Gongylosoma (Squamata: Colubridae) and description of a new Himalayan endemic genus and species
Source: Sci Rep. 2024 Oct 21;14:24743. doi: 10.1038/s41598-024-74271-1 (PMC11494134; doi:10.1038/s41598-024-74271-1)

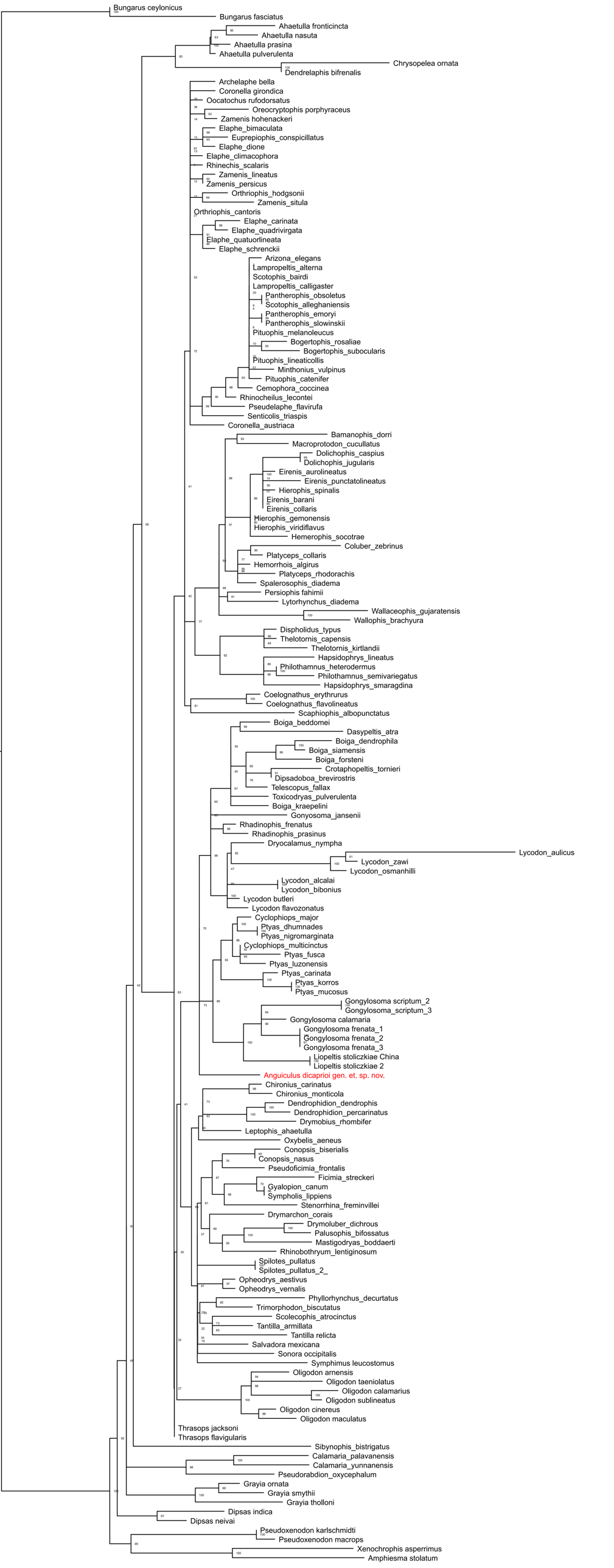

Supplement: Supplementary file 5 — Supplementary Material 5 [file 41598_2024_74271_MOESM5_ESM.pdf]

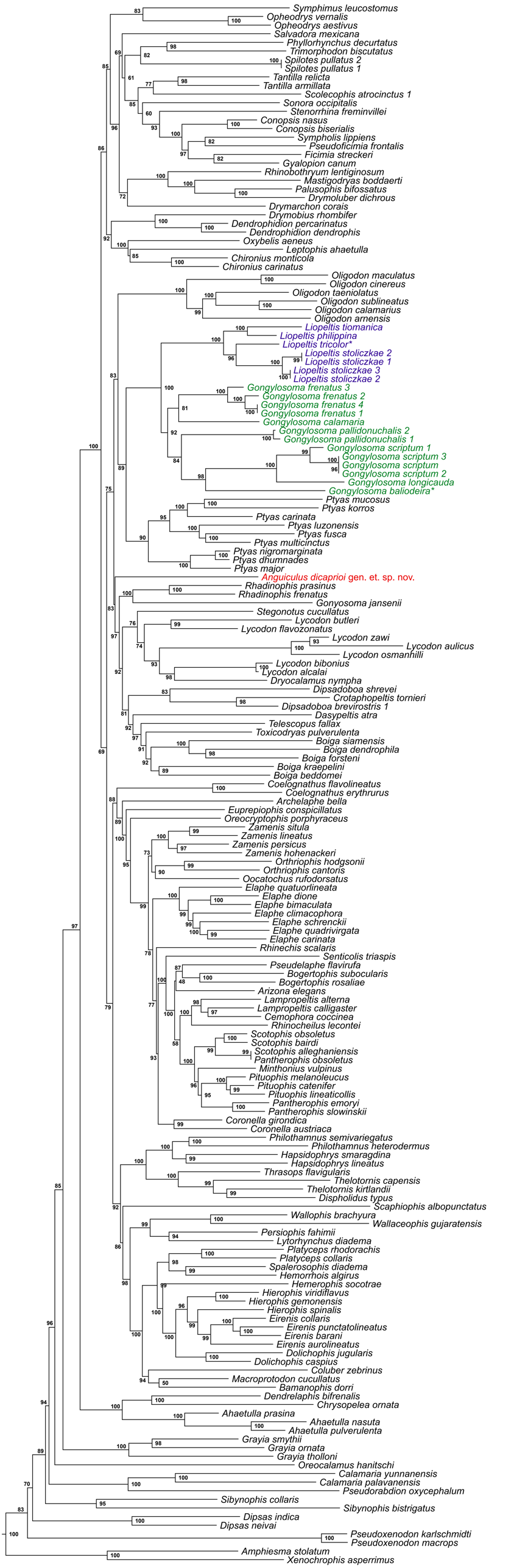

Supplement: Supplementary file 6 — Supplementary Material 6 [file 41598_2024_74271_MOESM6_ESM.pdf]
