# Supplementary material for: Phylogeny and systematics of the colubrid snake genera Liopeltis and Gongylosoma (Squamata: Colubridae) and description of a new Himalayan endemic genus and species
Source: Sci Rep. 2024 Oct 21;14:24743. doi: 10.1038/s41598-024-74271-1 (PMC11494134; doi:10.1038/s41598-024-74271-1)
Supplement: Supplementary file 7 — Supplementary Material 7 [file 41598_2024_74271_MOESM7_ESM.docx]

*
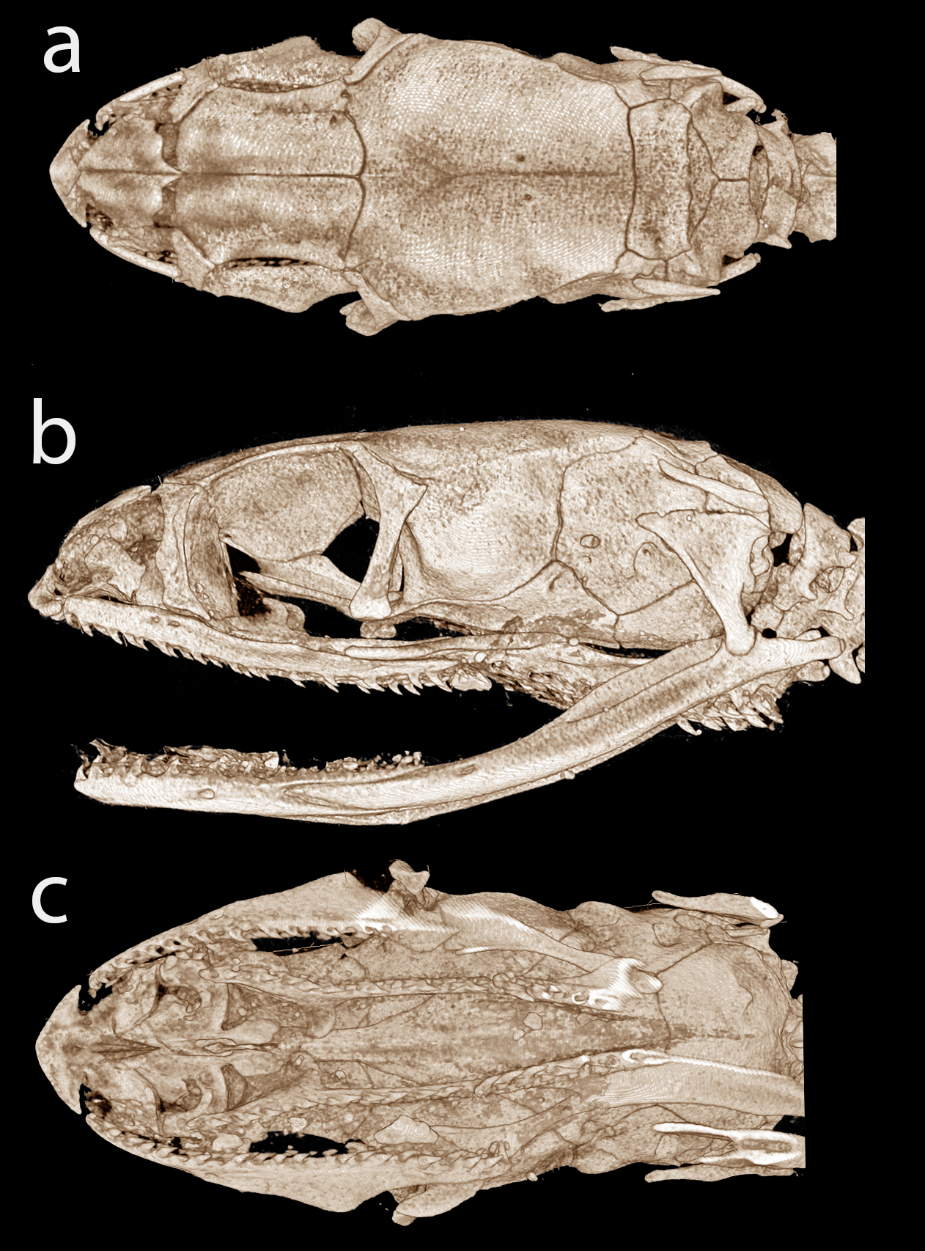
*

Figure S1. MicroCT images of the skull of *Gongylosoma calamaria* (BNHS 666), (a) dorsal view, (b) lateral view, (c) ventral view.


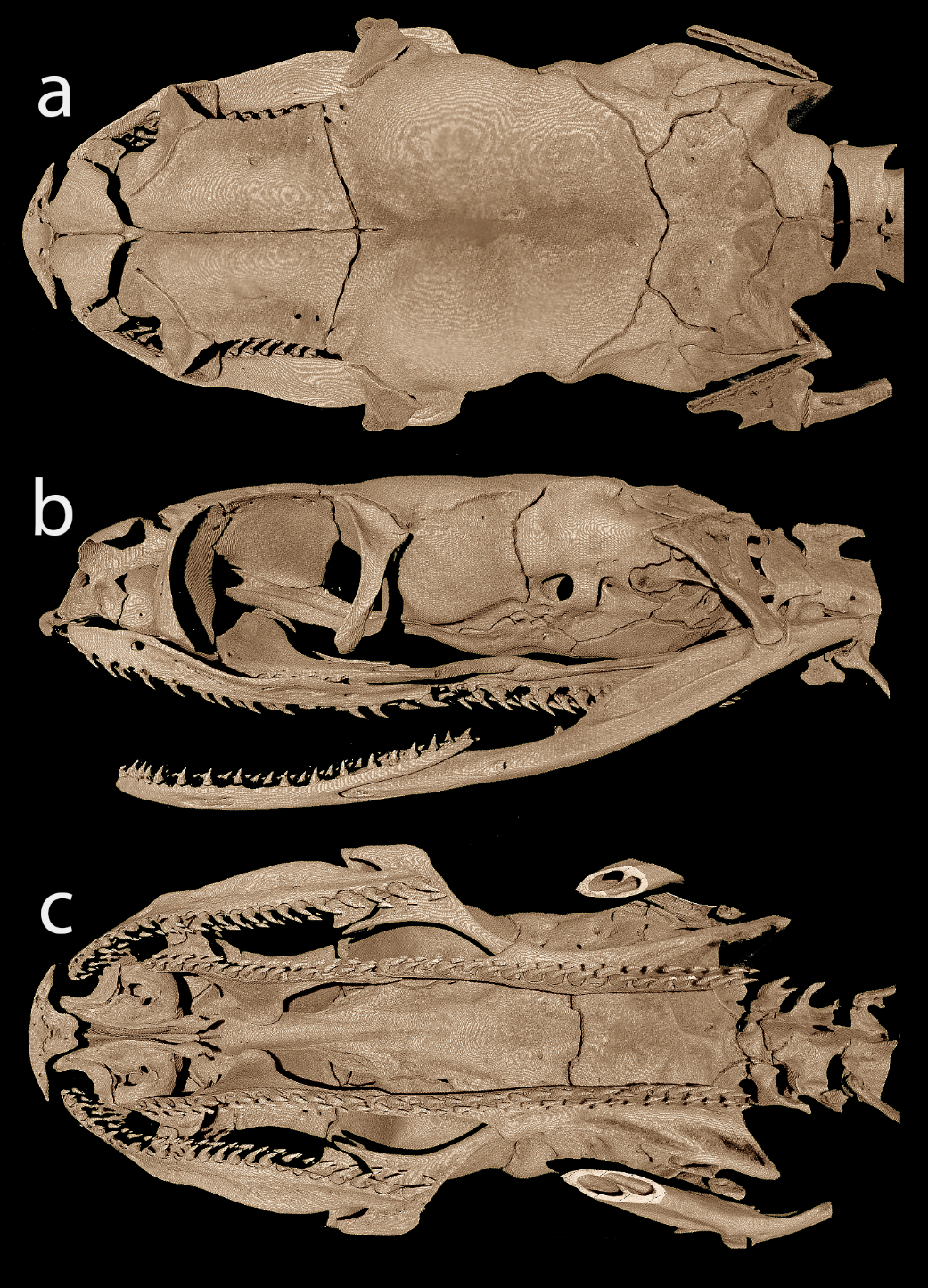


Figure S2. MicroCT images of the skull of *Gongylosoma scriptum* (MZMU 2041), (a) dorsal view, (b) lateral view, (c) ventral view.

*
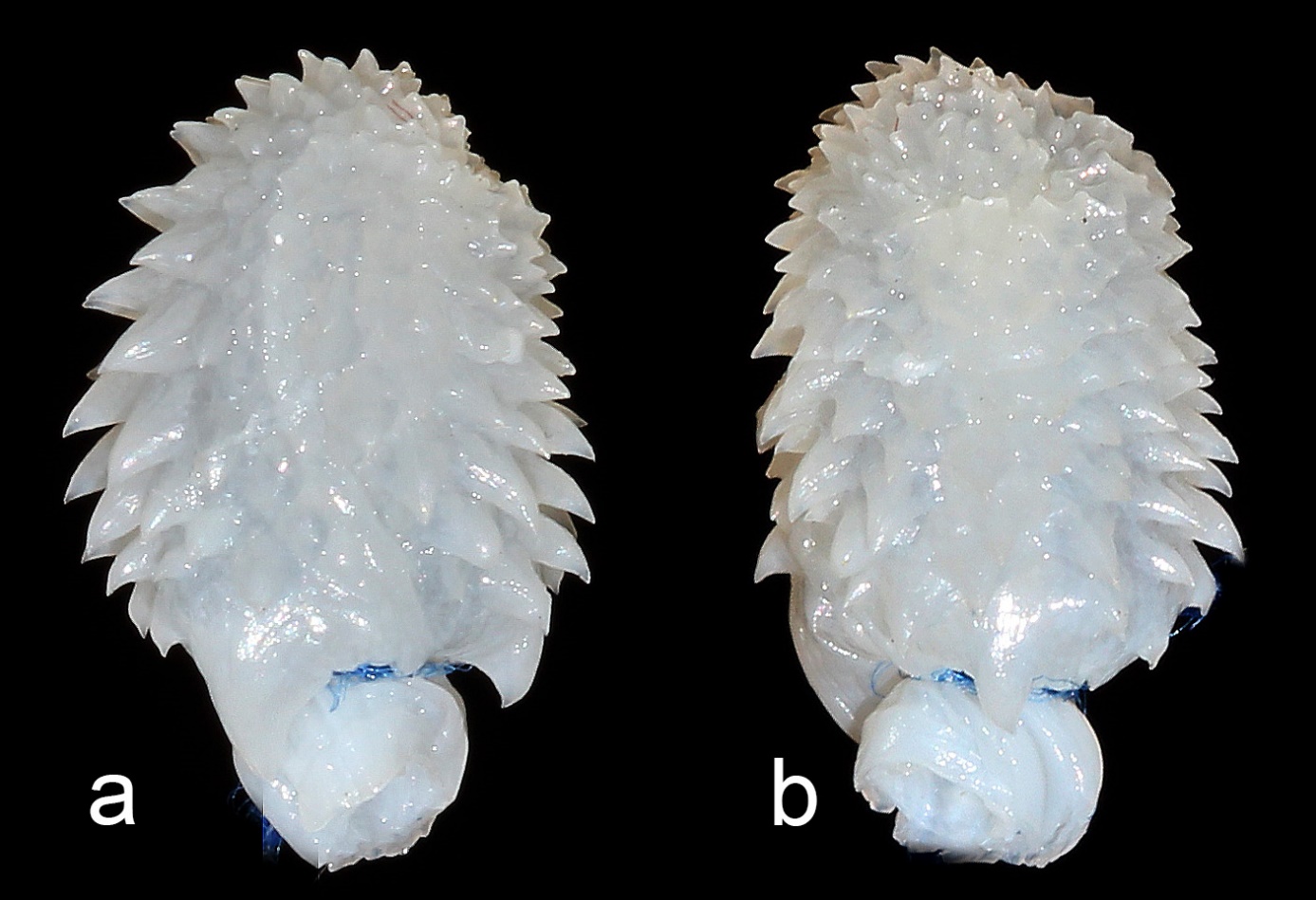
*

Figure S3. Hemipenis of *Anguiculus dicaprioi* **gen. et. sp. nov.**, (a) sulcul view, (b) asuclul view.

*
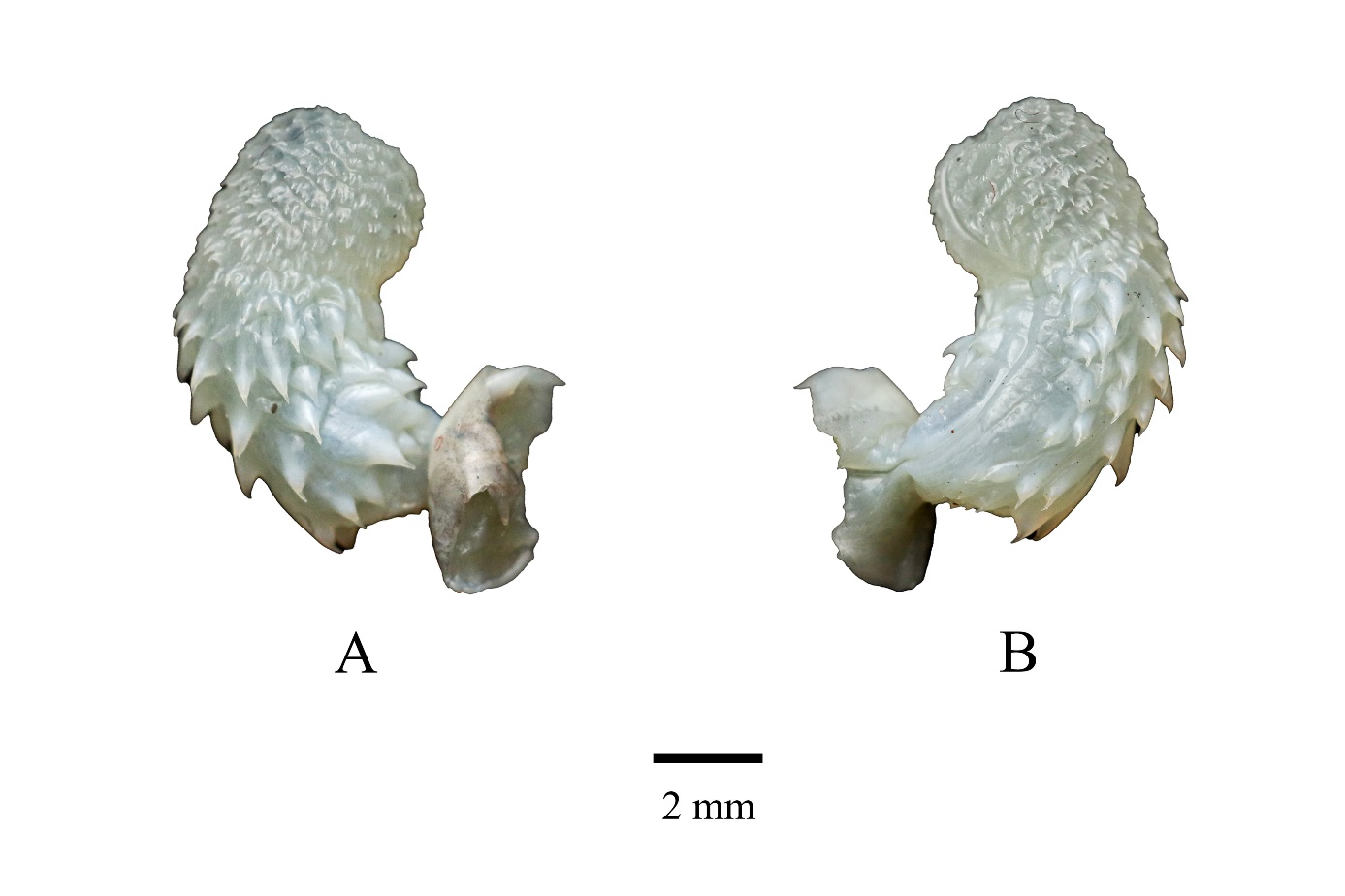
*

Figure S4. Hemipenis of *Gongylosoma frenata* MZMU965

*
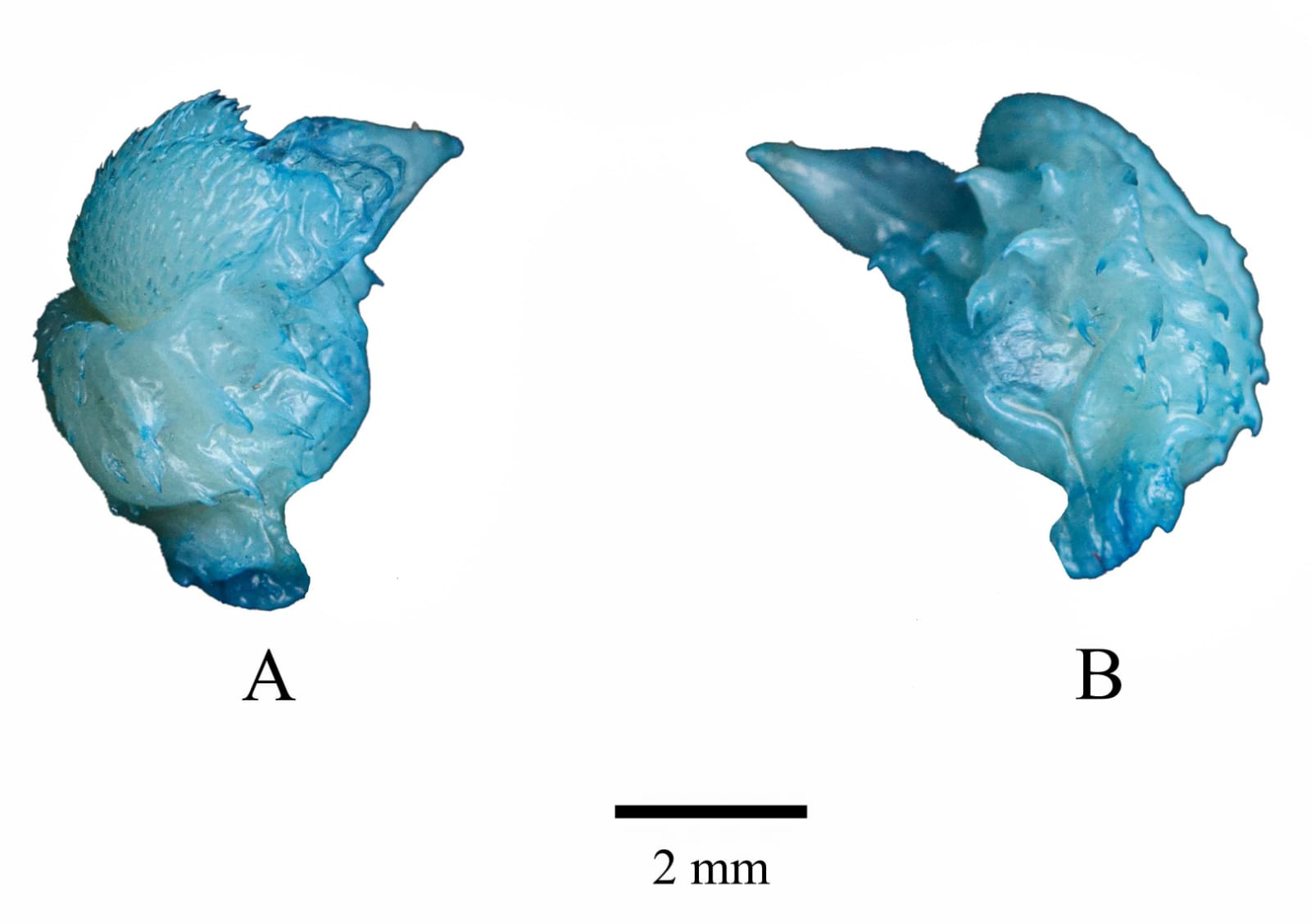
*

Figure S5. Hemipenis of *liopeltis stoliczkae* MZMU1597
